# Prevalent Glucocorticoid and Androgen Activity in US Water Sources

**DOI:** 10.1038/srep00937

**Published:** 2012-12-06

**Authors:** Diana A. Stavreva, Anuja A. George, Paul Klausmeyer, Lyuba Varticovski, Daniel Sack, Ty C. Voss, R. Louis Schiltz, Vicki S. Blazer, Luke R. Iwanowicz, Gordon L. Hager

**Affiliations:** 1 Laboratory of Receptor Biology and Gene Expression Building 41, B602 41 Library Dr. National Cancer Institute, NIH Bethesda, MD 20892-5055; 2 Natural Products Support Group SAIC-Frederick, Inc Frederick National Laboratory for Cancer Research Frederick, MD 21702; 3 USGS-BRD Leetown Science Center 11649 Leetown Road Kearneysville, WV 25430; 4 Current address: Department of Pharmacology UMDNJ-Robert Wood Johnson Medical School, Piscataway, New Jersey 08854

## Abstract

Contamination of the environment with endocrine disrupting chemicals (EDCs) is a major health concern. The presence of estrogenic compounds in water and their deleterious effect are well documented. However, detection and monitoring of other classes of EDCs is limited. Here we utilize a high-throughput live cell assay based on sub-cellular relocalization of GFP-tagged glucocorticoid and androgen receptors (GFP-GR and GFP-AR), in combination with gene transcription analysis, to screen for glucocorticoid and androgen activity in water samples. We report previously unrecognized glucocorticoid activity in 27%, and androgen activity in 35% of tested water sources from 14 states in the US. Steroids of both classes impact body development, metabolism, and interfere with reproductive, endocrine, and immune systems. This prevalent contamination could negatively affect wildlife and human populations.

An endocrine disruptor is an exogenous substance or a mixture that alters function(s) of the endocrine system and consequently causes adverse health effects in an intact organism, its progeny, or (sub)populations^1^. EDCs can cause adverse biological effects in animals and humans^1^,^2^. A Scientific Statement of the Endocrine Society^3^ postulates that EDCs have effects on reproduction, breast development and cancer, prostate cancer, neuroendocrinology, thyroid metabolism, obesity, and cardiovascular endocrinology. Contamination of water sources with EDCs poses a serious concern for human and animal health, and threatens the integrity of aquatic ecosystems^3^,^4^. Harmful effects of synthetic progestogens^5^,^6^ and especially of estrogenic water contaminants^7^,^8^,^9^ on fish reproduction have been recently documented. Other abnormalities such as an increased susceptibility to infections as a result of a weakened immune system are also observed and associated with fish kills from the Potomac river watershed^10^,^11^, suggesting a possible contamination with additional classes of EDCs. At present, the levels of other steroidal EDCs in the environment are not efficiently monitored and/or regulated. Chemical methods for detection of EDCs have revealed the presence of five classes of steroid hormones comprising estrogens, androgens, progestogens, glucocorticoids, and a mineralocorticoid in water sources in China^12^. Studies in the US have also revealed contamination with some of these classes of EDCs^5^,^6^,^7^,^8^,^9^,^13^. However, the presence of steroids such as glucocorticoids or mineralocorticoids was never studied. In addition, it is unclear whether the EDCs detected in water could elicit steroid-specific biological response(s) in mammalian systems. To address these questions, it is crucial to develop and implement high throughput and low cost methods for detection of EDCs in the environment. The need for such methods is well recognized in the field^14^. The existing chemical approaches are expensive, time-consuming and incompatible with a large-scale sample testing. Moreover, these approaches can only identify and quantify chemicals that were already included in the analysis schedules, thus limiting the screen to known EDCs. When the nature of the EDCs is unknown beforehand it will remain undetected. This represents a serious limitation of the existing chemical methods and hinders the discovery of novel EDCs.

## Results

**Figure 1:**
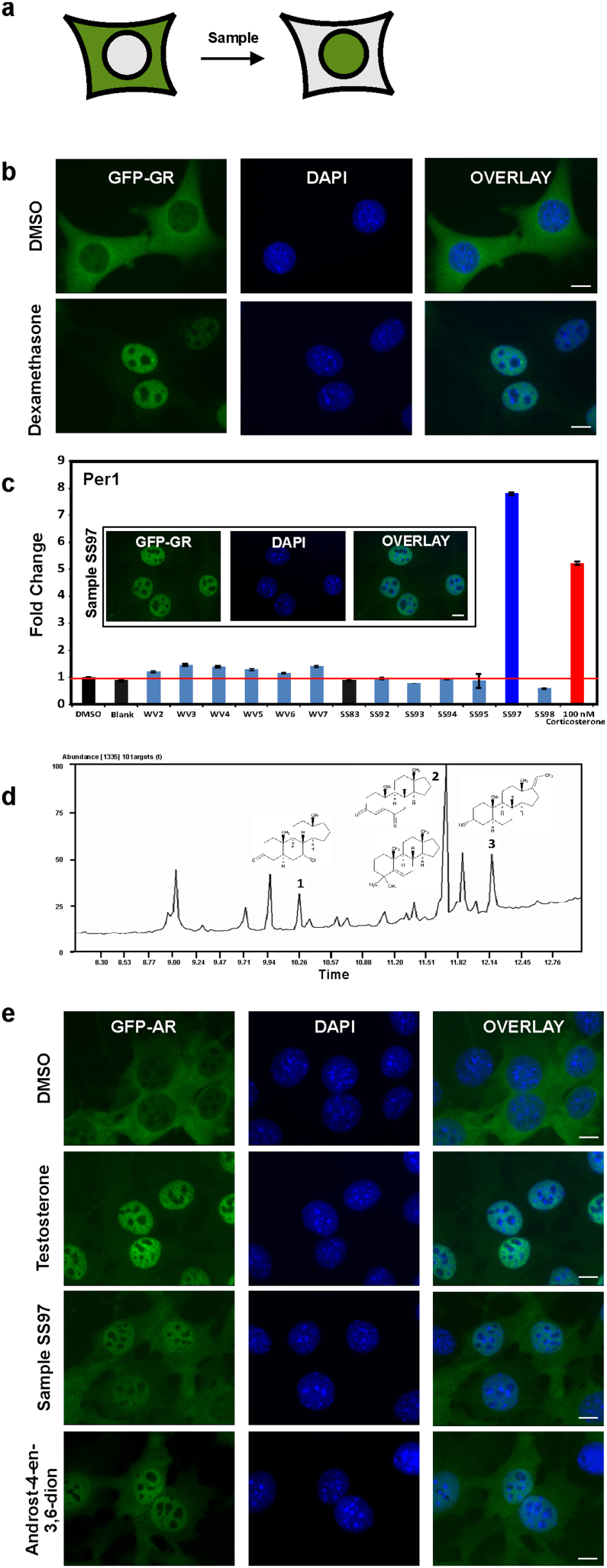
Analysis of water samples for glucocorticoid and androgen contamination. (a) Schematic representation of the GFP-tagged GR and AR receptor translocation in response to corresponding hormonal treatment. (b) GFP-GR translocation in a mammalian cell line^15^ upon stimulation with dexamethasone for 30 min. Nuclei are stained with DAPI. Scale bar, 5 μm. (c) Transcriptional activation of the GR-regulated Per1 gene by 10 water samples collected using a polar organic chemical integrative sampler (POCIS) are compared to transactivation induced by corticosterone. Data is normalized to DMSO alone. Blank and SS83 are POCIS negative controls. Error bars represent the mean ± s.e.m., n = 3. One of the water samples, SS97, induces complete GFP-GR translocation (image) and transcriptional activation of Per1 gene at a level higher than the activation induced by 100 nM corticosterone (graph). Scale bar, 5 μm. (d) GC/MS total ion chromatogram of HPLC fractionated sample SS97 (fraction 74-A) revealed the presence of a complex mixture of volatile hydrocarbons, as indicated by the peaks. Database searching of the extracted MS spectra corresponding to peaks 1–3 showed structural similarity to known androstane-type steroids. GC/MS analysis of these peaks is presented in Supplementary Fig. S2 and Table S2. (e) Representative images of GFP-AR nuclear translocation in response to 100 nM of testosterone, androst-4-ene-3,6-dione, and sample SS97 (100x). Scale bar, 5 μm.

Here we introduce a highly sensitive live cell assay based on cytoplasm to nuclear translocation of green fluorescent protein (GFP)-tagged nuclear receptors, exemplified by the glucocorticoid receptor (GR) expressed in a mammalian cell line, 3617^15^ to screen samples from US water sources for glucocorticoid/EDCs activity (Fig. 1a). This assay is based on the fact that, in the absence of corresponding hormone, GR resides in the cytoplasm bound to various heat-shock proteins and immunophilins in a large multi-protein complex^16^. Upon hormone binding, GR dissociates from this complex and translocates to the cell nucleus (Fig. 1a and b), where it interacts with GR regulatory elements (GREs) to elicit hormone-specific transcription regulation^17^. Naturally occurring glucocorticoids (GC) acting through glucocorticoid and mineralocorticoids receptors (GR and MR, respectively) are released in mammalian organisms in a complex circadian and ultradian manner^18^,^19^. Excess exposure to GC is associated with immune suppression and variety of other deleterious side effects^20^.

At present, the prevalence of GC activity in US water sources is unknown. However, using chemical methods, a few reports on water contamination have demonstrated detectable levels of glucocorticoids in the Netherlands and China^21^,^22^. Another recent study has demonstrated that environmentally relevant concentrations of synthetic GCs have deleterious effects on fish^23^. The anti-inflammatory properties of GCs make them highly prescribed pharmaceuticals which could readily enter water sources. Moreover, waste water treatment plants (WWTP) are not capable of efficiently removing GCs and it is well documented that anti-inflammatory chemicals are among the most resistant to treatment (30–40% of removal rate)^4^.

These data suggest a potential wide spread water contamination with GC activity at biologically-relevant concentrations. To directly examine this possibility, we tested 10 water samples collected from different locations (Supplementary Fig. S1a and Supplementary Table S1) for GC activity using GFP-GR translocation assay. The presence of GC activity in one of these samples (SS97) was revealed by the accumulation of GFP-GR in the nucleus within 30 minutes (Fig. 1c, images). To determine whether the detected GC activity can induce GR-mediated transcriptional response, we studied the transcriptional output of the Per1 gene upon activation with sample SS97 for 30 min. Per 1 is a direct GR-target gene^24^. It is a central part of the core transcriptional/translational feedback loop of the circadian oscillator. Exposure to exogenous glucocorticoids leads to untimely Per 1 activation and deregulation of the circadian oscillator implicated in the etiology of various diseases and metabolic disorders^25^. We discovered that sample SS97 induced Per1 to a significantly higher level than the positive control, corticosterone (at a physiologically relevant dose of 100 nM) (Fig. 1c, graph).

In an attempt to determine the active constituent(s) in sample SS97, known corticosteroids, dexamethasone and corticosterone, were tested by high performance liquid chromatography/mass spectrometry (HPLC/MS) analysis to establish chromatographic retention times on a C18 HPLC column^12^. In addition, 20 other synthetic GCs were surveyed by monitoring the mass spectrometric data for the presence of the corresponding molecular ions (Supplementary Table S2). Under these assay conditions, sample SS97 showed no evidence of any known compounds tested. Next, we subjected sample SS97 to HPLC fractionation followed by biological testing. Four of the eleven HPLC fractions showed activity in the nuclear translocation assay (Supplementary Fig. S1c). Again, when these fractions were tested by ultra performance liquid chromatography/mass spectrometry (UPLC/MS), no known GCs compounds were detected. The active fractions were also analyzed by gas chromatography/MS (GC/MS)^26^, and appeared similar in composition of volatile components. The mass spectra extracted from the GC/MS analysis were searched in both the NIST/EPA/NIH Mass Spectral Library 1998 and in the Wiley Mass Spectra Database of Androgens, Estrogens, and other Steroids 2010 (AES 2010), yielding no hits of high certainty for any of the peaks. However, visual comparison of the mass spectra of chromatographic peaks 1–3 (Fig. 1d) with standard spectra from the AES 2010 database (Supplementary Fig. S2) suggested similarities to known androstane-class compounds (Fig. 1d, Supplementary Table S3). One of these structures, androst-4-en-3,6-dione (peak 2), was synthesized^27^ and further tested for biological activity. Androst-4-en-3,6-dione did not induce GFP-GR translocation (data not shown) whereas it induced GFP-tagged androgen receptor (GFP-AR) translocation (Fig. 1e) using a GFP-AR expressing cell line, 3108^28^. These data suggest that, in addition to GCs, sample SS97 also contains androgenic activity.

Androgens are the original anabolic steroids and the precursor of all estrogens, the female sex hormones. Through their binding to androgen receptor (AR), they control the development and maintenance of male characteristics in vertebrates^29^. Similarly to the GR, AR is largely cytoplasmic in the absence of its ligand, and rapidly translocates to the nucleus in response to testosterone^28^ (Fig. 1e).

We conclude that environmental degradation and metabolic processes alter the structure of the glucocorticoid(s) in water samples, producing bioactive chemical structures which are not contained in the existing databases. Rapid transformation of hormonal steroids by aquatic microorganisms has been reported previously^30^. We also conclude that, in contrast to the traditional chemical analysis, the translocation assay is faster, cheaper, and also detects biologically relevant hormonal activity which cannot be discerned by chemical methods. Translocation assay allows unbiased “non-candidate” approach for detection of EDCs and could be used in a powerful combination with fractionation methods and “forensic chemistry” in the discovery of novel bioactive ligands.

**Figure 2:**
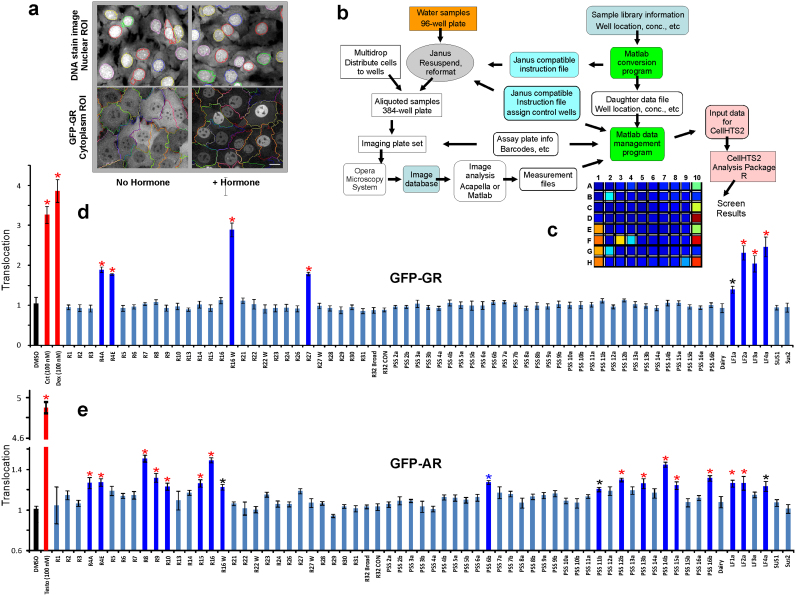
Water sample screening by high throughput automated image analysis. (a) Examples of images scored for cytoplasmic and nuclear segmentation from control and corticosterone treated 3617 cells. (b) Workflow for image-based screening of environmental contaminants with glucocorticoid activity using the Perkin Elmer Opera Image Screening System. (c) Automated image analysis output for a representative experiment. In this series, eight water samples from a total of 69 (well positions 2B, 2G, 3F, 4F, 9H, 10A, 10C, and 10E corresponding to samples R4E, R4A, R16W, R27, LF1a, 2a, 3a, and 4a (d, see below) tested positive for GFP-GR translocation as indicated by color changes. Wells 1A–D represent four negative (DMSO) controls. Wells 1E–H are positive controls for cells treated with 100 nM corticosterone, and wells 10D, 10F and 10H are positive controls for cells treated with 100 nM dexamethasone. (d) and (e) Quantitative analysis for GFP-GR and GFP-AR nuclear translocation, respectively. Translocation was calculated as a ratio of the nuclear versus cytoplasmic intensity, and each value was normalized to the control. Samples positive for glucocorticoid activity are marked with asterisks (P<0.01, red asterisks and P<0.05, black asterisks). Error bars represent the mean value ± s.e.m, n = 4.

**Figure 3:**
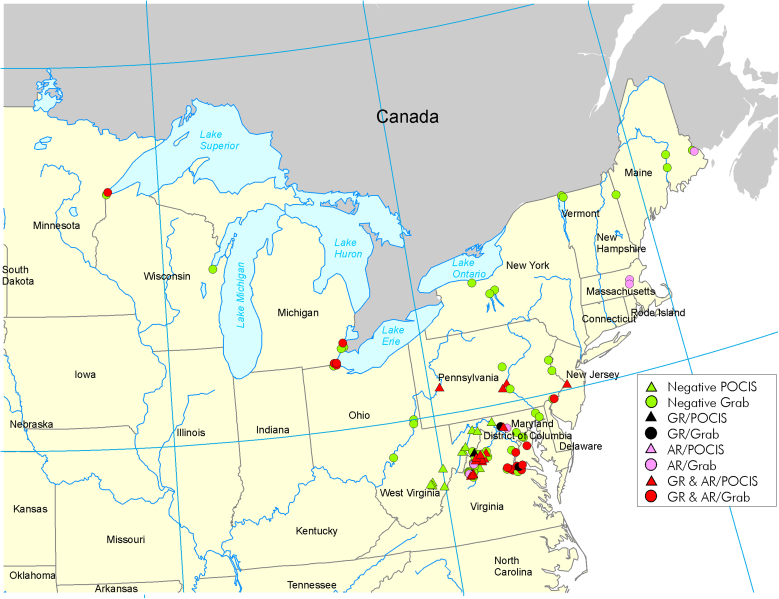
Geographic locations of the collection sites and their contamination with glucocorticoid and androgenic activity. Negative samples are marked with green color. Samples positive for glucocorticoid activity are marked with black, androgen activity-positive samples are marked with pink, and samples positive for both activities are marked with red. Triangles indicate grab samples, while the circles indicate the use of POCIS membranes. For complete sample description (collection method as well as the time of collection and translocation activity) see Supplementary Tables S4a and b.

Next, we expanded our search to screen over 100 additional samples from water sources throughout 14 states in the US (Supplementary Table S4a) for both, glucocorticoid and androgen activities. To accomplish this screening, we implemented the GFP-GR- and GFP-AR-expressing cell lines^15^,^28^ in an automated imaging analysis system (Perkin Elmer Opera Image Screening System) and used an algorithm for cytoplasm and nuclear segmentation to calculate translocation efficiency (Fig. 2a and b). To test the sensitivity and reproducibility of the automated assay, we measured translocation efficiency in response to known concentrations of the respective hormones. GFP-GR translocated to the nucleus in a concentration-dependent manner in response to the rodent, human, as well as synthetic hormones (corticosterone, hydrocortisone, and dexamethasone, respectively) (Supplementary Fig. S3a). The GFP-tagged AR also translocated to the nucleus in concentration-dependent manner in response to testosterone as well as synthesized androst-4-en-3,6-dione (Supplementary Fig. S3b and c). Confident in the sensitivity of the translocation assay, we tested the additional water samples which were divided into two plates: plate one [P1, (Fig. 2d, e)] and plate two [P2, (Supplementary Fig. S4 and Supplementary Fig. S5)]. We discovered that glucocorticoid activity was evident in over 28% (Fig. 2 d and Supplementary Fig. S4) and androgen activity in 37% (Fig. 2e and Supplementary Fig. S5) of the 105 samples subjected to the high throughput screening (Supplementary Tables S4a and b). When combined with the results obtained from the first manual screen of 10 samples (Supplementary Table S1), glucocorticoid and androgen activity remained in the same range (27% and 35%, respectively). Our results unambiguously demonstrate a wide spread contamination of the US water sources from 14 different states with both, glucocorticoid and androgenic activities (Fig. 3, Supplementary Tables S4a and b).

**Figure 4:**
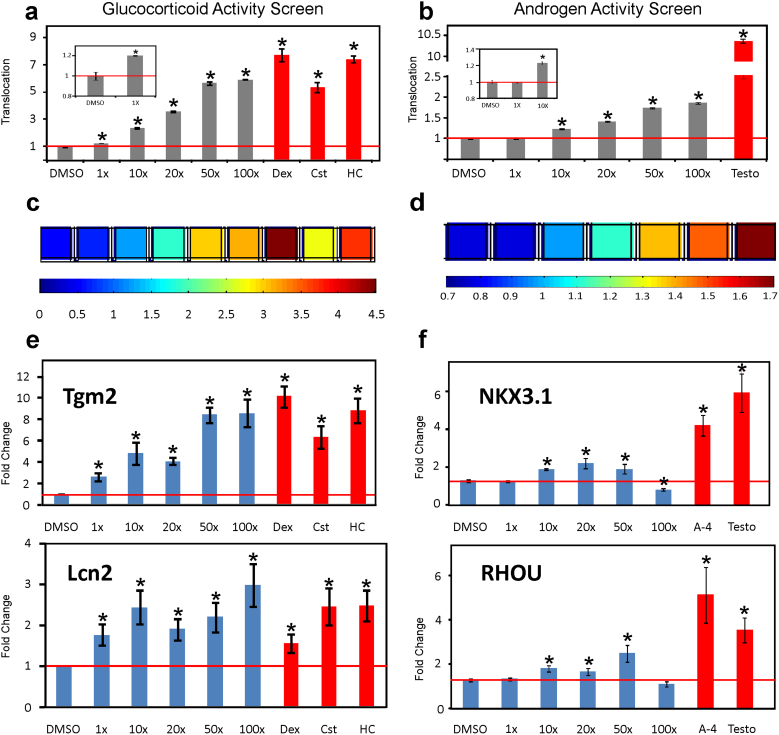
Concentration-dependent translocation and transcriptional activation induced by newly collected grab samples at location SS97. (a) and (b) Concentration-dependent GFP-GR and GFP-AR translocation for sample SS97 four years after the initial collection. Translocation is calculated from the automatic image analysis and expressed as a ratio of nuclear versus cytoplasmic intensity normalized to DMSO treated control. Samples positive for glucocorticoid and androgen activities are marked with asterisks (P<0.05). Error bars represent the mean value ± s.e.m, n = 4. Significant increase in the GFP-GR translocation is detectable in 1x dilution for this sample (inset). While the lowest concentration inducing significant GFP-AR translocation was 10x (b, insert). (c) Representative heat-map for concentration-dependent GFP-GR translocation indicated as nuclear versus cytoplasmic intensity. Dex (dexamethasone, 100 nM), Cst (corticosterone, 100 nM), and HC (hydrocortisone, 100 nM) are included as positive controls as indicated on the bar graph above (a). (d) Representative heat-map for the concentration-dependent GFP-AR translocation. Testosterone (Testo, 100 nM) was included as a positive control, as indicated on the bar graph above (b). (e) Concentration-dependent transcriptional activation of the GR-regulated genes, Tgm2 and Lcn2. All tested concentrations (including 1x) induced transcriptional responses of both genes, presented as fold change from the vehicle (DMSO) treated control. Dex (dexamethasone, 100 nM), Cst (corticosterone, 100 nM), and HC (hydrocortisone, 100 nM) are included as positive controls. Error bars represent the mean ± s.e.m, n = 4. (f) Concentration-dependent transcriptional activation of the AR-regulated genes, NKX3.1 and RHOU by sample SS97 in LNCaP cells. Gene transcription was induced by the concentrations 10x, 20x, and 50x (P<0.05, asterisks), whereas higher concentrations seemed to have an inhibitory activity. Data is presented as fold change in comparison to the vehicle (DMSO) treated control. Androst-4-en-3,6-dione (A-4) (100 nM) and testosterone (Testo, 100 nM) were included as positive controls. Error bars represent the mean ± s.e.m, n = 4.

Considering that the tested samples were collected over a span of several years (Supplementary Tables S1 and S4a), we sought to determine whether the observed contaminations persist over time. We revisited two of the previously identified contaminated sites (SS97 and GL2W) and collected grab water samples anew. As shown in Fig. 4a and b (as well as in Supplementary Figs. S6, S7, S8, S9) both newly collected samples induced GFP-GR and GFP-AR nuclear translocation in a concentration-dependent manner, suggesting high and persisting water contamination at these sites. Ten fold concentrated samples from both locations were active in GR and AR translocation assays, as well as induced transcriptional activity. Moreover, at 1x concentration, sample SS97 induced significant GFP-GR translocation (Fig. 4a-insert) and activation of gene transcription from the GR-responsive genes Tgm2 and Lcn2 (Fig. 4e). Both genes have been selected for their fast response to GCs, and have important physiological functions. Tgm2 has been implicated in inflammatory processes, such as Celiac disease, infections, cancer, neurodegenerative diseases, such as Huntington's disease and was recently found to be a key regulator of cross-talk between autophagy and apoptosis^31^. Lcn2 protein has been implicated in neuroinflammation by inducing CNS cells to secrete chemokines such as CXCL10, which recruit additional inflammatory cells^32^. Both approaches, the translocation assay as well as the GR-mediated transcriptional induction of GR-target genes indicate that the water at the SS97 location has biologically relevant glucocorticoid activity which is persistent over time.

## Discussion

Here we utilized mammalian cell lines expressing GFP-tagged nuclear receptor constructs in an automated, highly reproducible, and low cost assay for detection of biologically active glucocorticoids and androgens in US water sources. Using this high-throughput screening, combined with studies on transcriptional activation, we discovered glucocorticoid and androgen activities in POCIS and grab water samples from 8 of 14 states in the US. We utilized two AR-responsive genes, NKX3.1 and RHOU, to study the AR-dependent transcriptional activation by contaminated water samples. NKX3.1 is a homeobox gene required for prostate tumor progression and was implicated in androgen-dependent prostate cancer survival^33^, while RHOU was implicated in epidermal growth factor receptor signaling and cell migration^34^. Interestingly, 100x concentration of samples SS97 (Fig. 4f) and GL2W (Supplementary Fig. S9c) were less potent than the lower doses in inducing gene transcription from the two AR-regulated genes. This could be an example of the well known phenomenon of nonmonotonic dose-response where the effects of the low doses of EDCs cannot be predicted by the effects observed at high doses^35^,^36^. These results underscore the importance of examining the effects of a range of concentrations when using gene transcription analyses as readout for the biological effect of EDCs. However, presence of inhibitory components or antiestrogens in these samples cannot be ruled out and will require further investigation. In contrast, the GFP-GR and GFP-AR translocation assays were applicable to a wide range of concentrations including 100x doses. Thus, the translocation assay is largely devoid of the nonmonotonic dose-response effects observed by other detection methods, which makes it suitable for high-throughput screening.

We conclude that this level of wide-spread contamination with steroids of both classes is a possible health hazard not only for the aquatic ecosystems, but may also negatively impact the human population. Thus, aquatic organisms as well as other animals and humans exposed to contaminated water sources are subjected to atypical hormonal influences. It is possible that some of the effects of this exposure may not be immediately apparent but could influence the progeny during gestation and development. A growing body of epidemiological and animal studies suggests that prenatal and early life conditions contribute to health later in life. Exposures to stress hormones, including glucocorticoids, during the prenatal period have programming effects on the hypothalamic-pituitary-adrenal axis, brain neurotransmitter systems, and cognitive abilities of the offspring^37^. Prenatal exposure to endogenous or synthetic glucocorticoids is also associated with negative effects on the immune function of the offspring^38^. Disruption of androgen receptor signaling in males by environmental chemicals is also well documented^39^. Prenatal exposure of guinea pigs and monkeys to androgens is associated with irreversible changes in sexual and social behaviors later in life^40^,^41^. Thus, contamination of water sources with both glucocorticoid and androgenic activity could have long-term consequences.

Largely unrestricted human activity with respect to pharmaceuticals and other potential endocrine disruptors is of concern, and represents one of the main reasons for these wide-spread contaminations. In addition, limited methods for the detection of EDCs in the environment impair their efficient screening^14^. Our studies not only highlight the prevalence of contamination of water sources with glucocorticoid and androgen activities, but also introduce a novel approach for monitoring the quality of water. This approach could be readily extended to other nuclear receptors and applied to detection of various classes of EDCs in the environment.

## Methods

### Samples collection

Environmental water samples were collected as part of ongoing U.S. Geological Survey (USGS) projects that were implemented to monitor the presence and effects of endocrine-disruptors and other contaminants of emerging concern. They were collected between 2005 and 2010 from different geographic locations in the United States (Fig. 3), and included discrete grab water samples, or samples collected via polar organic chemical integrative samplers (POCIS). Samples in the R series were collected on or around National Wildlife Refuges in the Northeast and the GL series on tributaries of the Great Lakes, both were collected as part of collaborative projects with the US Fish and Wildlife Services. All other samples were collected as part of the USGS Chesapeake Bay Priority Ecosystems Science projects. Grab water samples were processed at the USGS, Leetown Science Center as described below.

#### POCIS samples

The POCIS membranes were shipped to the USGS, Columbia Environmental Research Center for analyte recovery. The procedures used for preparing the POCIS samples for analysis were described earlier^8^. Briefly, chemicals of interest were recovered from the POCIS sorbent using 50 mL of 1:1:8 (V:V:V) methanol:toluene:dichloromethane followed by 20 mL of ethyl acetate. The extracts were reduced by rotary evaporation, filtered, and composited into 2-POCIS equivalent samples thereby concentrating the amount of chemical present in each sample to aid in the detection.

#### Grab water samples

Grab water sample is defined as “a single sample or measurement taken at a specific time or over as short a period as feasible”. These samples were collected in 500 ml pre-cleaned amber glass bottles (I-Chem, Rockwood, TN). Water was acidified to pH3, held on ice, and stored at 4°C. Within one week of collection, the preserved water samples were filtered through a GF/F filter (0.7 ìm) using a solvent rinsed all-glass apparatus. Filters were rinsed with 1 ml of methanol to liberate soluble compounds from the retained suspended solids. Filtered samples and blanks were subjected to solid phase extraction (SPE) using OASIS® HLB (200 mg) glass cartridges (Waters Corporation, Milford, MA), following an existing protocol^42^. In short, cartridges were sequentially pre-conditioned and 400 ml of filtered samples were loaded onto the cartridge at a flow rate of 5–6 ml/minute (continuous vacuum). Analytes were eluted from the cartridge with 100% methanol and concentrated by rotary evaporation.

For biological testing, samples were reconstituted in DMSO and diluted in growth media to a final 1,000x concentration from the original water volume while maintaining DMSO at <0.2%. Samples were added to cells for 30 min at 100x concentration or as indicated in the text.

### Cell lines and translocation assay

The 3617 and 3108 cell lines are derivatives of 3134 mouse mammary adenocarcinoma cell line that express green fluorescent protein (GFP)-tagged GR (GFP-GR) and AR (GFP-AR), respectively from a chromosomal locus under control of the tetracycline-repressible promoter^15^,^28^. Prior to imaging, cells were grown overnight on 22-mm^2^ coverslips in DMEM medium containing 10% charcoal stripped serum (Hyclone, Logan, UT) without tetracycline (to allow the expression of the GFP-GR or GFP-AR, respectively) at a density of 2 × 10^5^ per 6-well plate. For the automated experiments conducted in 96 or 384 well plates, cell density was 10, 000 or 2,500 cells per well, respectively. Cells were treated with vehicle control, hormones (100 nM) or water samples for 30 min at 37°C at a final concentration of 100x for water samples (if not specified otherwise). Additional negative controls contained samples that tested the activity of the POCIS membranes themselves. Upon treatment, cells were fixed with 4% paraformaldehyde in PBS for 15 min and washed 3 times with PBS. Cells on the 22-mm^2^ coverslips were mounted in vectashield with DAPI (Vector Laboratories, Inc.) and examined on a Leica DMRA microscope with Leica 100x 1.3-N.A. oil immersion objective. Images were acquired in green (GFP-GR and GFP-AR) and UV (DAPI) channel with SenSys (Photometrics) camera with KAF1400 chip configured to collect 0.067-μm-diameter pixels. For the automated experiments conducted in 96 or 384 well plates, cells were stained with DRAQ5 (BioStatus Limited) at concentration 1:5000 for 15 min and after 3 final washes with PBS were imaged either immediately on the Perkin Elmer Opera Image Screening System or kept in PBS at 4°C for later imaging.

### Automated imaging and analysis by Perkin Elmer Opera Image Screening System

Perkin Elmer Opera Image Screening System was used for fully automated collection of images. This system employed a 40x water immersion objective lens, laser illuminated Nipkow disk, and cooled CCD cameras to digitally capture high resolution confocal fluorescence micrographs (300 nm pixel size with 2×2 camera pixel binning). An algorithm was customized using the Acapella image analysis software development kit (Perkin Elmer) to automatically segment both the nucleus and cytoplasm of each cell in the digital micrographs. The algorithm also measured the mean GFP-GR or GFP-AR intensity in both compartments, and translocation was calculated as a ratio of these intensities. Each value was further normalized to the value for the control (DMSO) sample.

### Gene transcription analysis

For gene transcription studies, 3134 cells or LNCaP cells (expressing endogenous GR and AR, respectively) were plated in 24-well dishes 24 h before experiment in DMEM (3134 cells) or RPMI (LNCaP cells) media supplemented with charcoal stripped fetal bovine serum (Hyclone, Logan, UT). Cells were treated with water samples, vehicle control (DMSO) or GR and AR specific hormones for 30 minutes. To prevent cell stress, these experiments were performed in a specially adapted incubator, allowing treatment under conditions of stable CO_2_ and temperature levels throughout the duration of an experiment. Cells were lysed in 600 μl of RLT buffer (with β -mercaptoethanol added) followed by syringe/needle shearing. Total RNA was extracted using the RNeasy Mini Kit (Qiagen), including a DNaseI digestion step (RNase free DNase Set, Qiagen). One μg of RNA was reverse transcribed (iScript cDNA Synthesis Kit, BioRad) in 20 μl reaction volume and 0.5 μl was used per Q-PCR reaction using SyBr green and Bio-Rad IQ system (Biorad, Hercules, CA). Primer sequences were designed to amplify nascent RNA (amplicons that cross an exon/intron boundary). The primer sequences are shown in Table 1. PCR was performed as recommended by the manufacturer. Standard curves were created by 10-fold serial dilution of template. The expression data from three or more independent experiments were normalized to the expression of a control gene β-Actin (3134 cells) and GAPDH (LNCaP cells), the mean values and SEM were calculated and displayed as a fold change in relation to the control (DMSO treated) sample.

### Statistical analyses

Data were analyzed using the statistical functions of IBM SPSS Statistics 19 and SigmaPlot 11 (SPSS Inc., Chicago, IL). From the repeated experiments, the mean value was calculated for each sample. The mean values were used in a one-way analysis of variance test. If a significant F-value of P < 0.05 was obtained, a Dunnett's multiple comparison versus the control group analysis was conducted.

## Author Contributions

D.A.S. designed and performed translocation and transcription experiments, analyzed data, prepared figures, and wrote the manuscript. A.A.G. performed translocation experiments and contributed to experiment design. P.K. performed chemical analyses, interpreted the data and provided expertise. L.V. subcloned the GFP-AR cell line, analyzed data, and contributed to the writing of the manuscript. D.S. performed translocation, transcription experiments and analyzed data. T.C.V. assisted the automated imaging and analysis by Perkin Elmer Opera Image Screening System. R.L.S. performed experiments and cell culture maintenance. V.S.B. provided water samples and expertise. L.R.I. contributed to the study design, provided water samples and information on their distribution. G.L.H. designed and supervised the study, contributed to the writing of the manuscript, and preparation of the figures. All authors discussed the results and commented on the manuscript.

## Supplementary Material

Supplementary Information Supplementary Information

## Figures and Tables

**Table 1:** Primer sequences for Q-PCR analysis

**Mouse cells (3134)**	**Sequence**
Per1 For	CTTCTGGCAATGGCAAGGACTC
Per1 Rev	CAGCATCATGCCATCATACACACA
Tgm2 For	TGTCACCAGGGATGAGAGACGG
Tgm2 Rev	TCCAAATCACACCTCTCCAGGAG
Lcn2 For	ACCTCTCATTTCTTGCAGTTCCG
Lcn2 Rev	CAGGATGGAGGTGACATTGTAGCT
β-Actin For	AGTGTGACGTTGACATCCGTA
β-Actin Rev	GCCAGAGCAGTAATCTCCTTCT
**Human cells (LNCaP)**	**Sequence**
hNKX3.1 For	TGACAGTGGGCTGTTTGTTC
hNKX3.1 Rev	AAGACCCCAAGTGCCTTTCT
hRHOU For	TTTCAAGGATGCTGGCTCTT
hRHOU Rev	GGCCTCAGCTTGTCAAATTC
GAPDH For	AAGGTGAAGGTCGGAGTCAAC
GAPDH Rev	GGGGTCATTGATGGCAACAATA

## Notes

**Patents:** A provisional patent covering this technology has been filed: U.S. Patent Application No. 61/656,473.

## References

[b1] WHO Global assessment of the state-of-the-science of endocrine disruptors. . WHO/PCS/EDC/02.2 (2011).

[b2] Diamanti-Kandarakis E., Palioura E., Kandarakis S. A. & Koutsilieris M. The impact of endocrine disruptors on endocrine targets. Horm. Metab Res 42, 543–552 (2010). 20419622 10.1055/s-0030-1252034

[b3] Diamanti-Kandarakis E. *et al.* Endocrine-disrupting chemicals: an Endocrine Society scientific statement. Endocr. Rev. 30, 293–342 (2009). 19502515 10.1210/er.2009-0002 PMC2726844

[b4] Deblonde T., Cossu-Leguille C. & Hartemann P. Emerging pollutants in wastewater: A review of the literature. Int. J Hyg. Environ. Health 214, 442–448 (2011). 21885335 10.1016/j.ijheh.2011.08.002

[b5] Zeilinger J. *et al.* Effects of synthetic gestagens on fish reproduction. Environ. Toxicol. Chem. 28, 2663–2670 (2009). 19469587 10.1897/08-485.1

[b6] Paulos P. *et al.* Reproductive responses in fathead minnow and Japanese medaka following exposure to a synthetic progestin, Norethindrone. Aquat. Toxicol. 99, 262 (2010). 10.1016/j.aquatox.2010.05.001 20617545

[b7] Iwanowicz L. R. *et al.* Reproductive health of bass in the Potomac, U.S.A., drainage: Part 1. Exploring the effects of proximity to wastewater treatment plant discharge. Environ. Toxicol. Chem. 28, 1072–1083 (2009). 19102584 10.1897/08-433.1

[b8] Alvarez D. A. *et al.* Reproductive health of bass in the Potomac, U.S.A., drainage: Part 2. Seasonal occurrence of persistent and emerging organic contaminants. Environ. Toxicol. Chem. 28, 1084–1095 (2009). 19108592 10.1897/08-417.1

[b9] Blazer V. S. *et al.* Reproductive endocrine disruption in smallmouth bass (Micropterus dolomieu) in the Potomac River basin: spatial and temporal comparisons of biological effects. Environ. Monit. Assess. 184, 4309–4334 (2012). 21814719 10.1007/s10661-011-2266-5 PMC3374114

[b10] Ripley J., Iwanowicz L., Blazer V. & Foran C. Utilization of protein expression profiles as indicators of environmental impairment of smallmouth bass (Micropterus dolomieu) from the Shenandoah River, Virginia, USA. Environ. Toxicol. Chem. 27, 1756–1767 (2008). 18315392 10.1897/07-588

[b11] Blazer V. S. *et al.* Mortality of centrarchid fishes in the Potomac drainage: survey results and overview of potential contributing factors. J Aquat. Anim Health 22, 190–218 (2010). 21192549 10.1577/H10-002.1

[b12] Chang H., Wan Y. & Hu J. Determination and source apportionment of five classes of steroid hormones in urban rivers. Environ. Sci. Technol. 43, 7691–7698 (2009). 19921880 10.1021/es803653j

[b13] Kolpin D. W. *et al.* Pharmaceuticals, hormones, and other organic wastewater contaminants in U.S. streams, 1999–2000: a national reconnaissance. Environ. Sci. Technol. 36, 1202–1211 (2002). 11944670 10.1021/es011055j

[b14] Roy P. & Pereira B. M. A treatise on hazards of endocrine disruptors and tool to evaluate them. Indian J Exp. Biol 43, 975–992 (2005). 16313061

[b15] Walker D., Htun H. & Hager G. L. Using inducible vectors to study intracellular trafficking of GFP-tagged steroid/nuclear receptors in living cells. Methods (Companion to Methods in Enzymology) 19, 386–393 (1999). 10.1006/meth.1999.0874 10579933

[b16] Pratt W. B. & Toft D. O. Steroid receptor interactions with heat shock protein and immunophilin chaperones. Endocr. Rev. 18, 306–360 (1997). 9183567 10.1210/edrv.18.3.0303

[b17] John S. *et al.* Interaction of the glucocorticoid receptor with the global chromatin landscape. Mol. Cell 29, 611–624 (2008). 18342607 10.1016/j.molcel.2008.02.010

[b18] Lightman S. L. Patterns of exposure to glucocorticoid receptor ligand. Biochem. Soc. Trans. 34, 1117–1118 (2006). 17073764 10.1042/BST0341117

[b19] Stavreva D. A. *et al.* Ultradian hormone stimulation induces glucocorticoid receptor-mediated pulses of gene transcription. Nat. Cell Biol. 11, 1093–1102 (2009). 19684579 10.1038/ncb1922 PMC6711162

[b20] Schacke H., Docke W. D. & Asadullah K. Mechanisms involved in the side effects of glucocorticoids. Pharmacol. Ther. 96, 23–43 (2002). 12441176 10.1016/s0163-7258(02)00297-8

[b21] Schriks M. *et al.* High-resolution mass spectrometric identification and quantification of glucocorticoid compounds in various wastewaters in the Netherlands. Environ. Sci. Technol. 44, 4766–4774 (2010). 20507090 10.1021/es100013x

[b22] Chang H., Hu J. & Shao B. Occurrence of natural and synthetic glucocorticoids in sewage treatment plants and receiving river waters. Environ. Sci. Technol. 41, 3462–3468 (2007). 17547164 10.1021/es062746o

[b23] Kugathas S. & Sumpter J. P. Synthetic glucocorticoids in the environment: first results on their potential impacts on fish. Environ. Sci. Technol. 45, 2377–2383 (2011). 21322550 10.1021/es104105e

[b24] Balsalobre A. *et al.* Resetting of circadian time in peripheral tissues by glucocorticoid signaling. Science 289, 2344–2347 (2000). 11009419 10.1126/science.289.5488.2344

[b25] Chung S., Son G. H. & Kim K. Circadian rhythm of adrenal glucocorticoid: its regulation and clinical implications. Biochim. Biophys. Acta 1812, 581–591 (2011). 21320597 10.1016/j.bbadis.2011.02.003

[b26] Mansilha C. *et al.* Quantification of endocrine disruptors and pesticides in water by gas chromatography-tandem mass spectrometry. Method validation using weighted linear regression schemes. J Chromatogr. A 1217, 6681–6691 (2010). 20553685 10.1016/j.chroma.2010.05.005

[b27] Hunter A. C. & Priest S. M. An efficient one-pot synthesis generating 4-ene-3, 6-dione functionalised steroids from steroidal 5-en-3beta-ols using a modified Jones oxidation methodology. Steroids 71, 30–33 (2006). 16183090 10.1016/j.steroids.2005.07.007

[b28] Klokk T. I. *et al.* Ligand-specific dynamics of the androgen receptor at its response element in living cells. Mol. Cell Biol. 27, 1823–1843 (2007). 17189428 10.1128/MCB.01297-06 PMC1820481

[b29] Gottlieb B., Lombroso R., Beitel L. K. & Trifiro M. A. Molecular pathology of the androgen receptor in male (in)fertility. Reprod. Biomed. Online. 10, 42–48 (2005). 15705293 10.1016/s1472-6483(10)60802-4

[b30] Yin G. G., Kookana R. S. & Ru Y. J. Occurrence and fate of hormone steroids in the environment. Environ. Int. 28, 545–551 (2002). 12503920 10.1016/s0160-4120(02)00075-2

[b31] Rossin F., D'Eletto M., Macdonald D., Farrace M. G. & Piacentini M. TG2 transamidating activity acts as a reostat controlling the interplay between apoptosis and autophagy. Amino. Acids 42, 1793–1802 (2012). 21479826 10.1007/s00726-011-0899-x

[b32] Lee S. *et al.* Lipocalin-2 Is a chemokine inducer in the central nervous system: role of chemokine ligand 10 (CXCL10) in lipocalin-2-induced cell migration. J. Biol. Chem. 286, 43855–43870 (2011). 22030398 10.1074/jbc.M111.299248 PMC3243551

[b33] Tan P. Y. *et al.* Integration of regulatory networks by NKX3-1 promotes androgen-dependent prostate cancer survival. Mol. Cell Biol. 32, 399–414 (2012). 22083957 10.1128/MCB.05958-11 PMC3255774

[b34] Zhang J. S., Koenig A., Young C. & Billadeau D. D. GRB2 couples RhoU to epidermal growth factor receptor signaling and cell migration. Mol. Biol. Cell 22, 2119–2130 (2011). 21508312 10.1091/mbc.E10-12-0969 PMC3113775

[b35] Vandenberg L. N. *et al.* Hormones and Endocrine-Disrupting Chemicals: Low-Dose Effects and Nonmonotonic Dose Responses. Endocr. Rev. (2012). 10.1210/er.2011-1050 PMC3365860 22419778

[b36] Fagin D. Toxicology: The learning curve. Nature 490, 462–465 (2012). 23099381 10.1038/490462a

[b37] Darnaudery M. & Maccari S. Epigenetic programming of the stress response in male and female rats by prenatal restraint stress. Brain Res. Rev. 57, 571–585 (2008). 18164765 10.1016/j.brainresrev.2007.11.004

[b38] Merlot E., Couret D. & Otten W. Prenatal stress, fetal imprinting and immunity. Brain Behav. Immun. 22, 42–51 (2008). 17716859 10.1016/j.bbi.2007.05.007

[b39] Luccio-Camelo D. C. & Prins G. S. Disruption of androgen receptor signaling in males by environmental chemicals. J. Steroid Biochem. Mol. Biol. 127, 74–82 (2011). 21515368 10.1016/j.jsbmb.2011.04.004 PMC3169734

[b40] Thornton J., Zehr J. L. & Loose M. D. Effects of prenatal androgens on rhesus monkeys: a model system to explore the organizational hypothesis in primates. Horm. Behav. 55, 633–645 (2009). 19446080 10.1016/j.yhbeh.2009.03.015 PMC3146061

[b41] Wallen K. & Hassett J. M. Sexual differentiation of behaviour in monkeys: role of prenatal hormones. J. Neuroendocrinol. 21, 421–426 (2009). 19207815 10.1111/j.1365-2826.2009.01832.x PMC2704567

[b42] Ciparis S., Iwanowicz L. R. & Voshell J. R. Effects of watershed densities of animal feeding operations on nutrient concentrations and estrogenic activity in agricultural streams. Sci. Total Environ. 414, 268–276 (2012). 22088420 10.1016/j.scitotenv.2011.10.017

