# Supplementary material for: Prevalent Glucocorticoid and Androgen Activity in US Water Sources
Source: Sci Rep. 2012 Dec 6;2:937. doi: 10.1038/srep00937 (PMC3515810; doi:10.1038/srep00937)
Supplement: Supplementary Information [file srep00937-s1.pdf]

Supplementary Information

Prevalent Glucocorticoid and Androgen Activity in US Water Sources

Authors : Diana A. Stavreva[1,*], Anuja A. George[1,4], Paul Klausmeyer[2], Lyuba Varticovski[1], Daniel Sack[1], Ty C. Voss[1], R. Louis Schiltz[1], Vicki S. Blazer[3], Luke R. Iwanowicz[3,*], and Gordon L. Hager[1,*]

Affiliations:

[1] Laboratory of Receptor Biology and Gene Expression, National Cancer Institute, NIH, Bethesda, MD 20892-5055

[2] Natural Products Support Group, SAIC-Frederick, Inc., Frederick National Laboratory for Cancer Research, Frederick, MD 21702

[3] USGS-BRD, Leetown Science Center, 11649 Leetown Road, Kearneysville, WV 25430

[4] Present address: Department of Pharmacology, UMDNJ-Robert Wood Johnson Medical School, Piscataway, New Jersey 08854

[*]To whom all correspondence should be addressed: hagerg@exchange.nih.gov
stavrevd@mail.nih.gov
luke_iwanowicz@usgs.gov

Supplementary Information Content: Supplementary Figures S1-9
Supplementary Tables S1-4

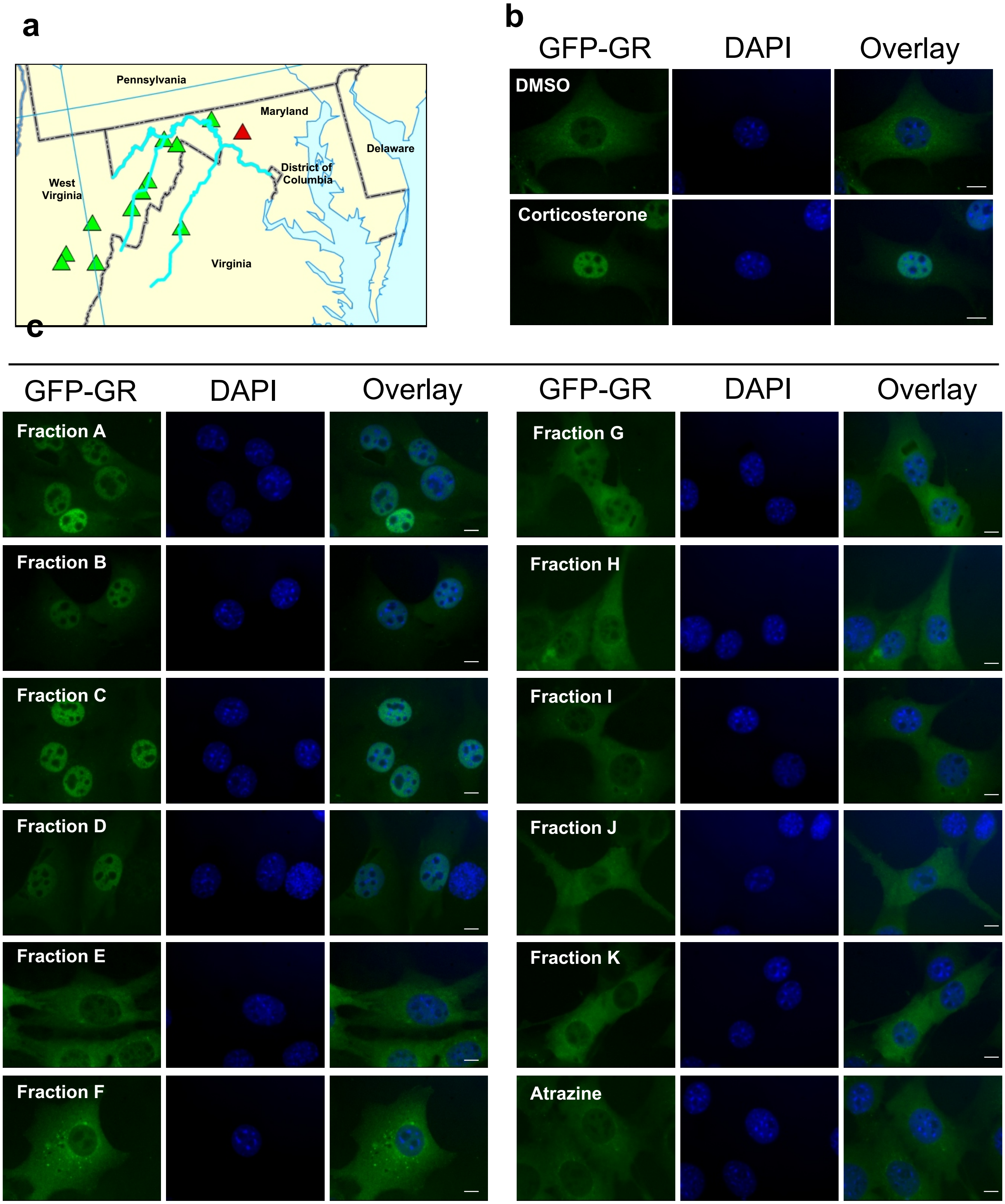

Supplementary Figure S1. Collection sites of the first sample set and testing of HPLC fractions from sample SS97 for GR translocation. (a) Geographic locations of the collection sites of the first sample set collected by POCIS. One of the samples (marked with red, SS97) tested positive for GFP-GR translocation, suggesting a presence of glucocorticoid activity. Negative samples are marked with green. (b) GFP-GR translocation in a mammalian cell line[15] upon stimulation with 100 nM corticosterone for 30 min. Nuclei are stained with DAPI. Scale bar, 5 µm. (c) HPLC fractions of sample SS97 (A-K) were tested for GFP-GR translocation to determine the presence of glucocorticoid activity. Four of 11 fractions (A-D) tested positive for GFP-GR translocation. Because GC/MS analyses detected atrazine in sample SS97, we included it in our analysis (bottom right). However, atrazine did not induce GFP-GR translocation.

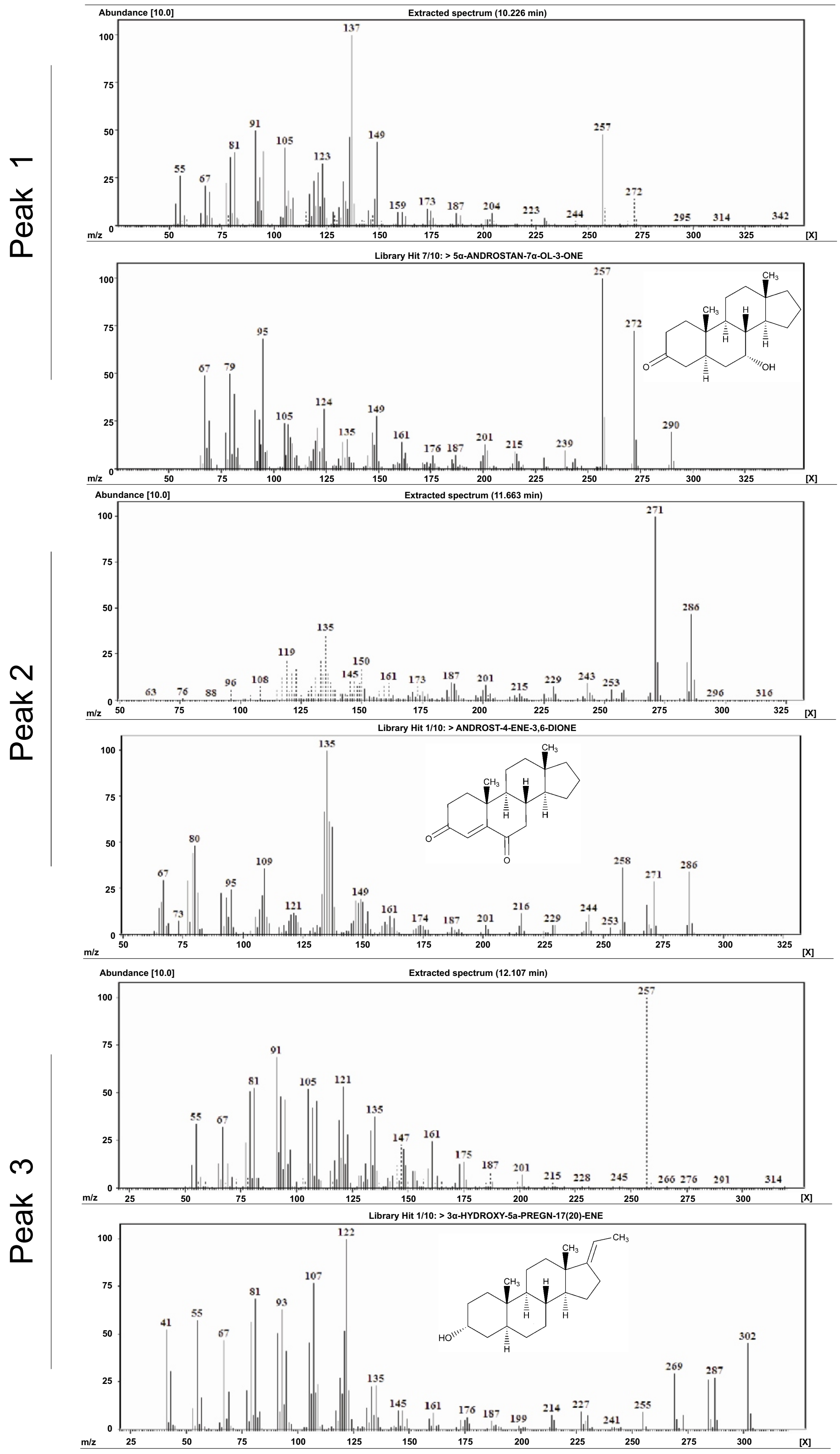

Supplementary Figure S2. Database searching of the extracted MS chromatographic peaks 1-3 (Fig. 1d) suggest that the compounds were similar in structure to known androstane-type steroids. Visual comparison of the mass spectra of chromatographic peaks 1-3 (Fig. 1d) with standard spectra from the AES 2010 database suggests similarities to known androstane-class compounds.

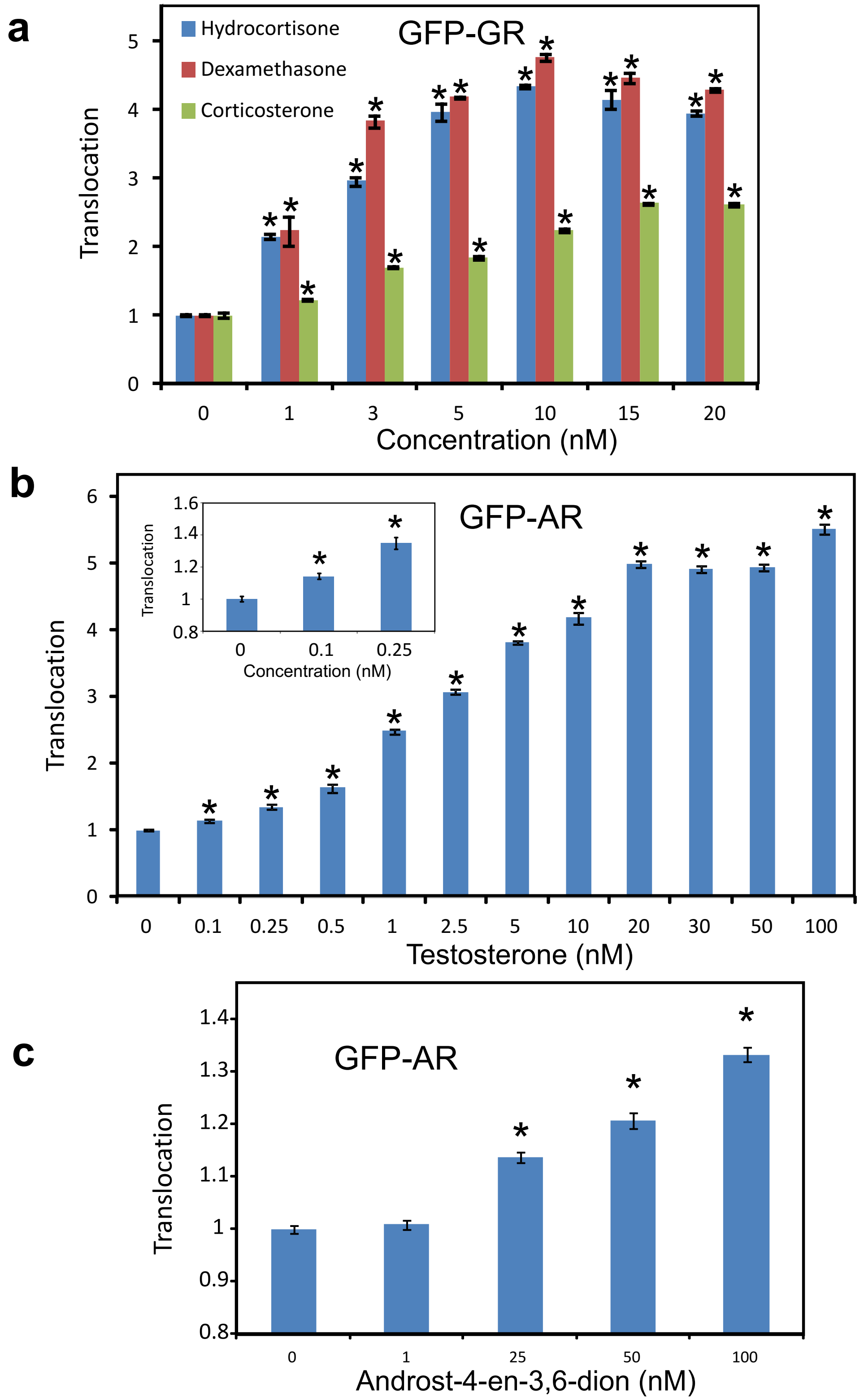

Supplementary Figure S3. Concentration-dependent translocation of GFP-GR and GFP-AR in response to their respective hormones as detected by the Opera (Perkin Elmer) automated imaging analysis system. (a) GFP-GR translocates to the nucleus in a concentration-dependent manner upon treatment with known concentrations of hydrocortisone, dexamethasone, or corticosterone. An algorithm for cytoplasm and nuclear segmentation of the cells was used to determine the mean GFP-GR intensity in both compartments and translocation was quantified as a ratio of these intensities. Each value was normalized to the control sample. Error bars represent the mean value ± s.e.m, n=6 ($P<0.05$, asterisks). (b) GFP-AR translocation in response to known concentrations of testosterone. Insert shows that testosterone concentrations as low as 0.1 nM induced a statistically significant increase in the GFP-AR translocation. Samples with $P<0.05$ are indicated by an asterisk. Error bars represent the mean value ± s.e.m, n=6. (c) Androst-4-en-3,6-dione induces concentration-dependent translocation of the GFP-AR construct ($P<0.05$, asterisks). Error bars represent the mean value ± s.e.m, n=6.

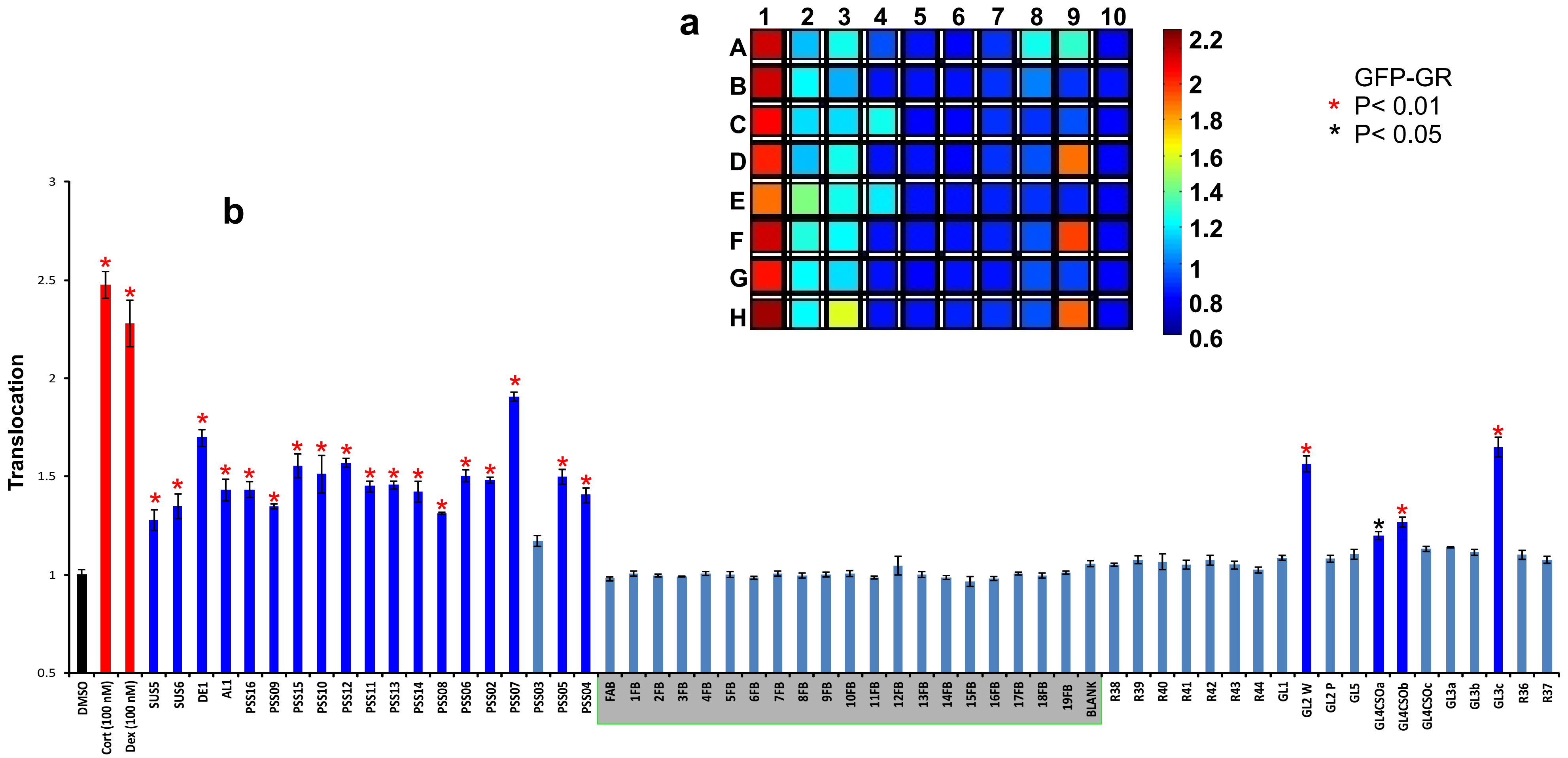

Supplementary Figure S4. Additional samples screened for GFP-GR nuclear translocation. (a) Image analysis plate map showing a portion of GFP-GR expressing cells plated on a 384 well plate. Twenty two samples out of 38 (58 %) tested positive for GFP-GR nuclear translocation. Wells 10A-H are negative controls (DMSO). Wells 1A-H and 9D, 9F, 9H are positive controls treated with 100 nM corticosterone and 100 nM dexamethasone, respectively. (b) GFP-GR nuclear translocation results summary. All samples in the gray box are POCIS negative controls. Positive samples are marked with asterisks (P<0.01, red asterisks and P<0.05, black asterisks). Error bars represent the mean value ± s.e.m, n=4.

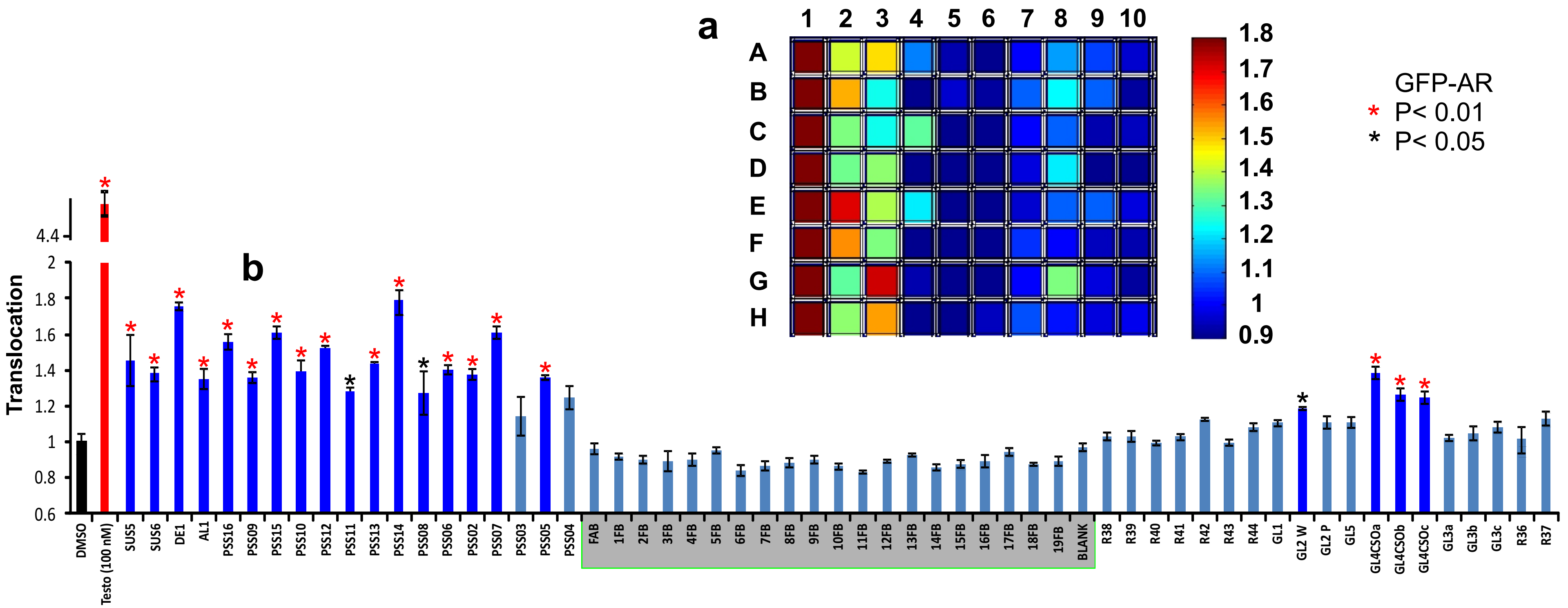

Supplementary Figure S5. Additional samples screened for GFP-AR nuclear translocation. (a) Image analysis plate map showing a portion of GFP-AR expressing cells plated on a 384 well plate. Twenty one samples out of 40 (55 %) tested positive for GFP-AR nuclear translocation. Wells 10A-H are negative controls (DMSO). Wells 1 A-H are positive controls for cells treated with 100 nM testosterone. (b) GFP-AR nuclear translocation results summary. All samples in the gray box are POCIS negative controls. Positive samples are marked with asterisks ($P<0.01$, red asterisks and $P<0.05$, black asterisks). Error bars represent the mean value ± s.e.m, n=4.

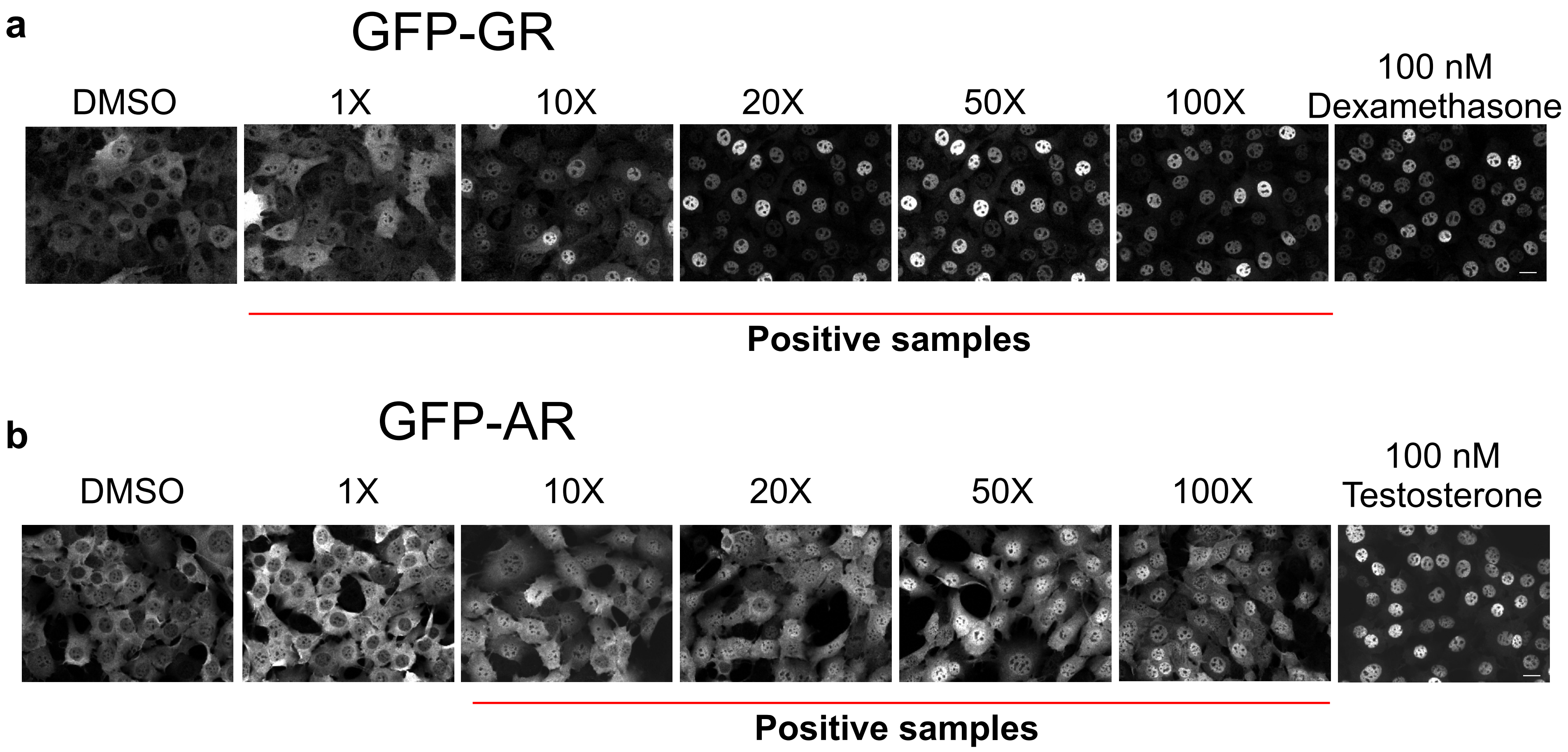

Supplementary Figure S6. Concentration-dependent GFP-GR and GFP-AR translocation induced by the newly collected sample from the same location as SS97. (a) Representative images for the concentration-dependent GFP-GR translocation in response to known concentrations of the water sample at site SS97. Scale bar, 10 μm. (b) Representative images for concentration-dependent GFP-AR translocation in response to known concentrations of the water sample at site SS97. Scale bar, 10 μm.

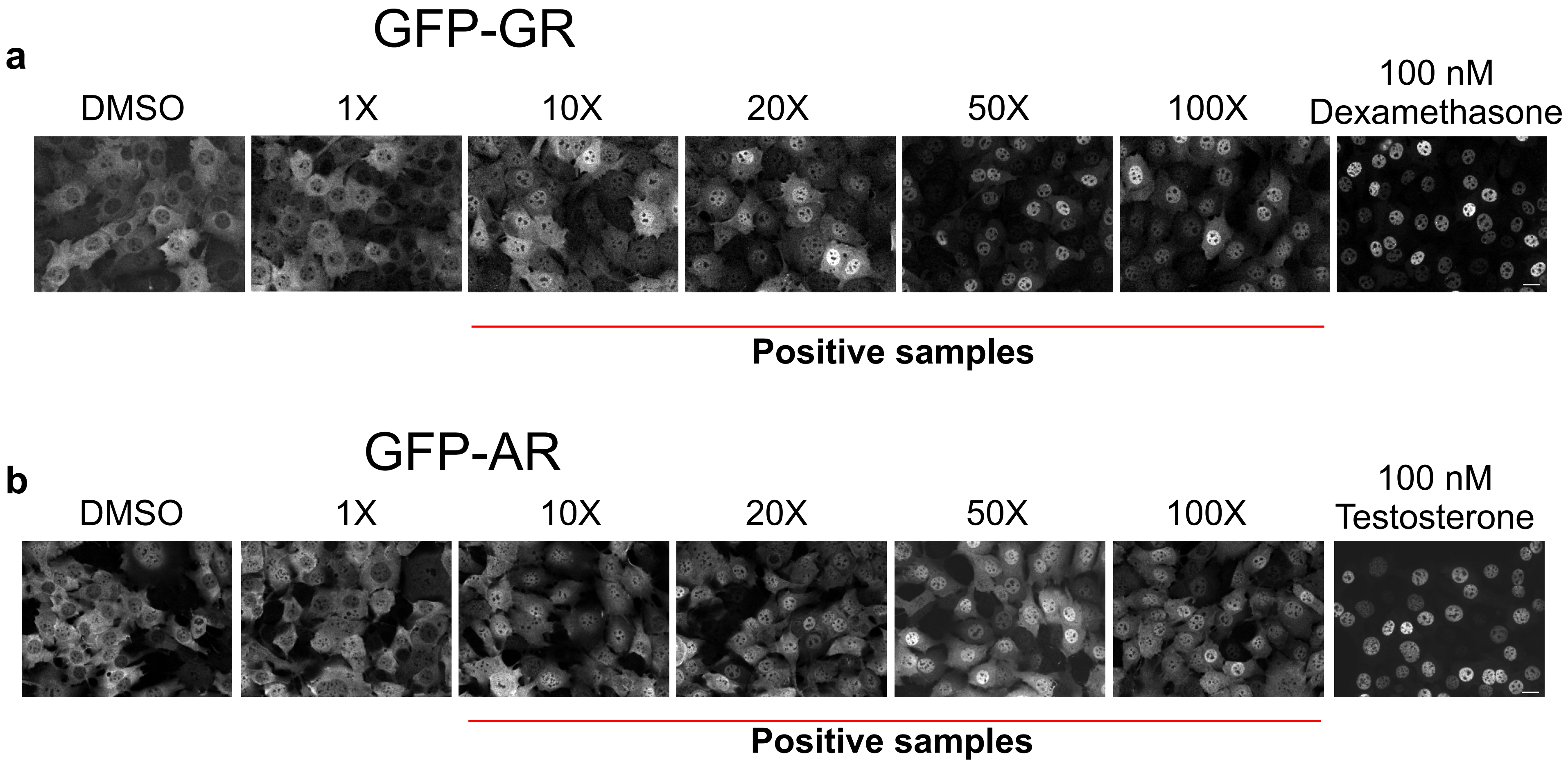

Supplementary Figure S7. Concentration-dependent GFP-GR and GFP-AR translocation induced by the newly collected sample from the same location as GL2W. (a) Representative images for concentration-dependent GFP-GR translocation in response to known concentrations of the water sample at site GL2W. Scale bar, 10 μm.(b) Representative images for the concentration-dependent GFP-AR translocation in response to known concentrations of the water sample at site GL2W. Scale bar, 10 μm.

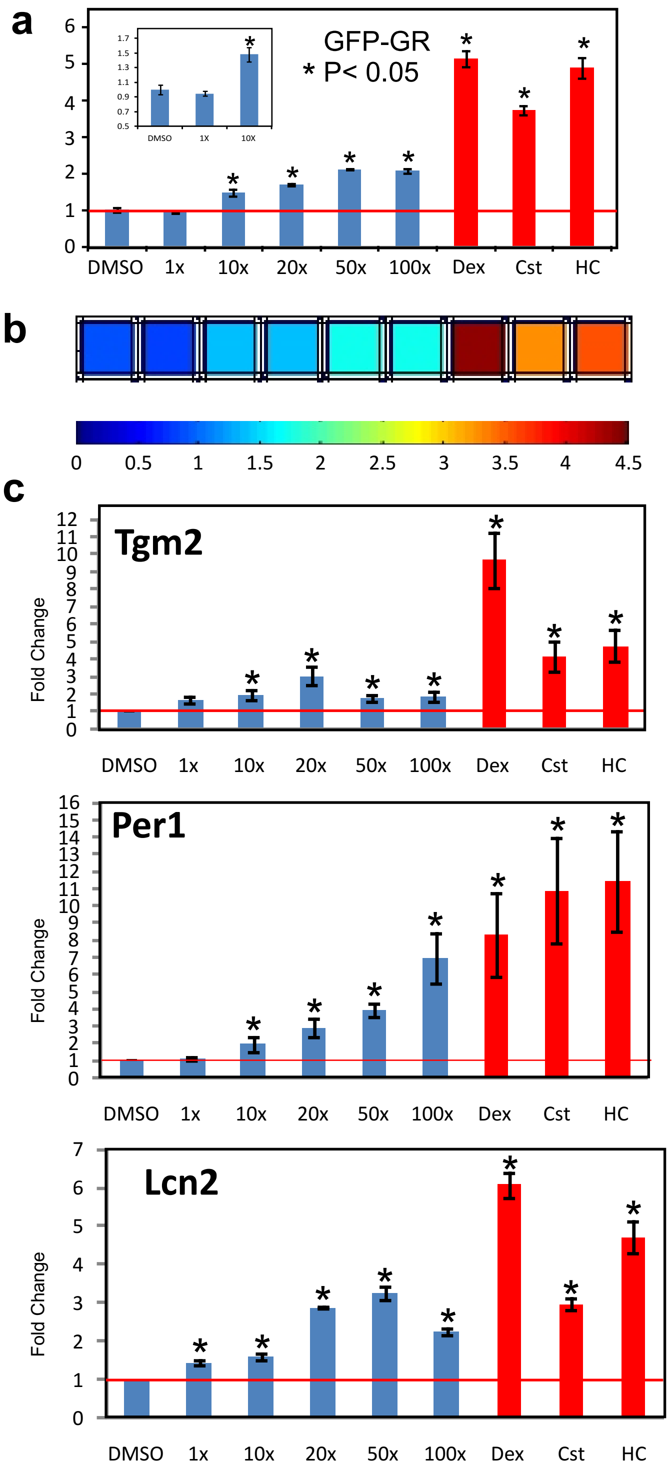

Supplementary Figure S8. Concentration-dependent GFP-GR translocation and transcriptional activation of GR-regulated genes in response to the newly collected sample from the same location as GL2W. (a) Translocation of GFP-GR in response to newly collected sample from the same location as GL2W (see also Supplementary Fig. S7a). Translocation was calculated as a ratio of the nuclear versus cytoplasmic intensity and each value was further normalized to the value for the control sample. Samples positive for GFP-GR translocation are marked with asterisks (P<0.05). The lowest concentration inducing GFP-GR translocation is 10x. DMSO negative control, Dex (dexamethasone, 100 nM), Cst (corticosterone, 100 nM), and HC (hydrocortisone, 100 nM) were included as positive controls. Error bars represent the mean value ± s.e.m, n=4. (b) Representative raw data heat-map for GFP-GR translocation as in panel A. (c) Concentration-dependent transcriptional activation of GR-regulated genes induced by newly collected sample from GL2W site. All concentrations induced transcriptional activation of at least one of the GR-regulated genes (P<0.05, asterisks). Transcription responses are presented as fold change in comparison to the vehicle control sample (DMSO) . Dex (dexamethasone, 100 nM), Cst (corticosterone, 100 nM), and HC (hydrocortisone, 100 nM) are included as positive controls. Error bars represent the mean ± s.e.m, n=4

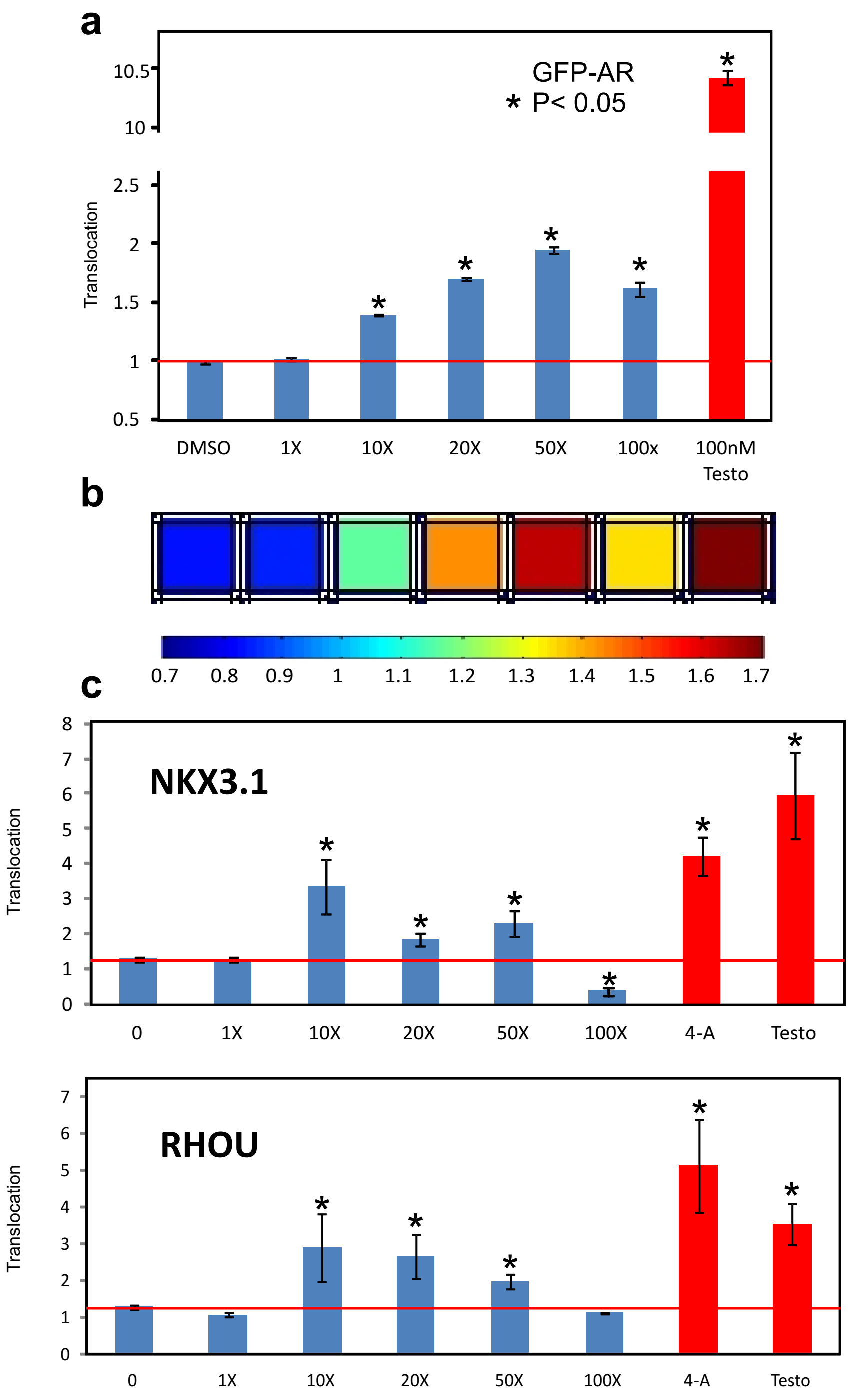

Supplementary Figure S9. Concentration-dependent GFP-AR translocation and transcriptional activation of AR-regulated genes in response to the newly collected sample from GL2W site. (a) Quantification of the GFP-AR concentration-dependent translocation response for the newly collected water sample at the GL2W site (see also Supplementary Fig. S7b). Translocation was calculated as a ratio of the nuclear versus cytoplasmic intensity and each value was further normalized to the value for the control sample. Samples positive for GFP-AR translocation are marked with asterisks (P<0.05). The lowest sample concentration inducing GFP-AR translocation is 10x. Testosterone (Testo, 100 nM) is included as a positive control. Error bars represent the mean value ± s.e.m, n=4. (b) Representative raw data heat-map for GFP-AR translocation as in panel (a). (c) Concentration-dependent transcriptional activation of AR-regulated genes induced by newly collected sample from GL2W site in LNCaP cells which express endogenous AR. Concentrations 10x to 50x induced transcriptional activation in the AR-regulated genes NKX3.1 and RHOU (P<0.05, asterisks). At 100x concentration, we observed a reduced NKX3.1 expression, and did not detect change in RHOU transcription, suggesting a nonmonotonic dose-response or the presence of an inhibitory activity (possibly anti-androgenic) which is apparent at that concentration. Transcriptional responses are presented as fold change in comparison to the vehicle treated (DMSO) control sample. Androst-4-en-3,6-dione (100 nM) and testosterone (Testo, 100 nM) are included as positive controls. Error bars represent the mean ± s.e.m, n=4.

Supplementary Table S1. Collection sites and collection time of the first sample set.

Samples	Date/year	Location	Collection method
Blank	Oct-Nov 2005		POCIS
WV2	Oct-Nov 2005	South Branch Potomac, Franklin, WV	POCIS
WV3	Oct-Nov 2005	South Bracnh Potomac, Springfield, WV	POCIS
WV4	Oct-Nov 2005	South Bracnh Potomac, Moorefield, WV	POCIS
WV5	Oct-Nov 2005	Elk River, WV	POCIS
WV6	Oct-Nov 2005	South Branch Potomac, Petersburg Gap, WV	POCIS
WV7	Oct-Nov 2005	Greenbrier River, WV	POCIS
SS83	6/1/2007		POCIS
SS92	5/3/07-6/7/07	Gauley River, WV	POCIS
SS93	4/11/07-5/9/07	Lower Conococheague River, MD	POCIS
SS94	4/11/07-5/9/07	Upper Conococheague River, MD	POCIS
SS95	4/27/07-5/31/07	South Branch Potomac, Petersburg Gap, WV	POCIS
SS97	4/11/07-5/9/07	Lower Monocacy River, MD	POCIS
SS98	4/5/07-5/9/07	South Fork, Shanandoah River, VA	POCIS

Supplementary Table S2. Synthetic glucocorticoids surveyed by monitoring the mass spectrometric data for the presence of the corresponding molecular ion in sample SS97.

1	amcinonide
2	betamethasone
3	budesonide
4	clobetasone
5	clobetasol
6	propionate
7	desonide
8	fluocinonide
9	fluocinolone acetonide
10	fluocortolone
11	fluprednidene acetate
12	halcinonide
13	hydrocortisone
14	hydrocortisone-17-butyrate
15	methylprednisolone
16	mometasone
17	mometasone furoate
18	prednicarbate
19	prednisolone
20	prednisone
21	triamcinolone acetonide

Supplementary Table S3. Closest EIMS library matches of the mass spectra of chromatographic peaks 1-3 in Fig. 1d.

Peak #	Observed MW	Closest EIMS Library Match	Structure
**1**	**272**	7α-Hydroxy-5α-androstan-3-one,<br>MW 290	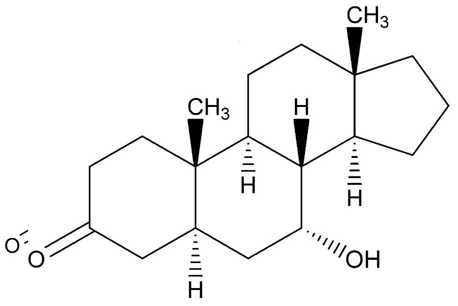
**2**	**286**	Androst-4-en-3,6-dione,<br>MW 286	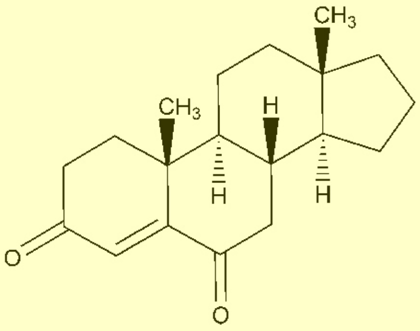
		or<br>4,4-Dimethyl-androst-5-ene,<br>MW 286	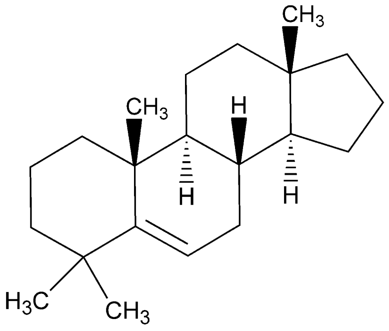
**3**	**272?**	3α-Hydroxy-17-ethylidene-5α-androstane,<br>MW 286	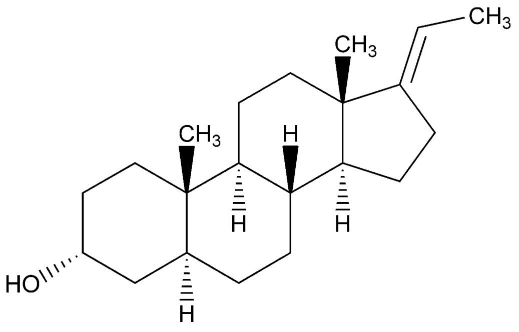

Supplementary Table S4. Additional samples tested for GFP-GR and GFP-AR translocation efficiency.

(a) Geographic location, time, and methods of collection of the water samples.

Samples	Date/year	Location	Collection method
Plate 1			
R1	9/2/2008	Ohio River, Wheeling WV	Grab sample
R2	9/3/2008	Ohio River, Parkersburg, WV	Grab sample
R3	9/3/2008	Upstream Brighton Dam, Tridelphia resevoir, MD	Grab sample
R4A	9/10/2008	Patuxent/Western Branch, MD	Grab sample
R4E	9/10/2008	Patuxent/Western Branch, MD (WWTP effluent)	Grab sample
R5	9/15/2008	Seneca River, NY	Grab sample
R6	9/16/2008	Seneca River, NY	Grab sample
R7	9/22/2008	St. Croix River, ME	Grab sample
R8	9/23/2008	St. Croix River, ME	Grab sample
R9	10/2/2008	Sudbury river, MA	Grab sample
R10	10/3/2008	Sudbury river, MA	Grab sample
R13	10/15/2008	Rappahanock River, VA	Grab sample
R14	10/15/2008	Rappahanock River, VA	Grab sample
R15	10/6/2008	Rappahanock River, VA	Grab sample
R16	10/27/2008	Darby Creek, PA	Grab sample
R16W	10/27/2008	Darby Creek, PA (WWTP effluent)	Grab sample
R21	9/1/2009	Missisquoi River, VT	Grab sample
R22	9/1/2009	Missisquoi River, VT	Grab sample
R22W	9/1/2009	Missisquoi River, VT (WWTP effluent)	Grab sample
R23	9/14/2009	Penobscot River, ME	Grab sample
R24	9/15/2009	Penobscot River, ME	Grab sample
R26	9/22/2009	Peconic Lake, NY	Grab sample
R27	9/28/2009	Potomac River, Pohick, VA	Grab sample
R27W	9/28/2009	Potomac River, Pohick, VA (WWTP effluent)	Grab sample
R28	9/29/2009	Burke Lake, VA	Grab sample
R29	10/5/2009	Delaware River, Cherry Valley, DE	Grab sample
R30	10/6/2009	Delaware River, Easton, PA	Grab sample
R31	10/13/2009	Susquehana River, Garret Island, PA	Grab sample
R32 Broad	10/14/2009	Susquehana River, Conowingo Dam, PA	Grab sample
R32 CON	10/14/2009	Susquehana River, Conowingo Dam, PA	Grab sample
PSS2a	5/14/2010	Gooney Run, VA	Grab sample
PSS2b	6/14/2010	Gooney Run, VA	Grab sample
PSS3a	5/14/2010	Passage Creek, VA	Grab sample
PSS3b	6/15/2010	Passage Creek, VA	Grab sample
PSS4a	5/14/2010	Stony Creek (upstream), VA	Grab sample
PSS4b	6/15/2010	Stony Creek (upstream), VA	Grab sample
PSS5a	5/14/2010	Stony Creek (downstream), VA	Grab sample
PSS5b	6/15/2010	Stony Creek (downstream), VA	Grab sample
PSS6a	5/13/2010	Mill Creek, VA	Grab sample
PSS6b	6/14/2010	Mill Creek, VA	Grab sample
PSS7a	5/14/2010	Hawksbill Creek, VA	Grab sample
PSS7b	6/14/2010	Hawksbill Creek, VA	Grab sample

PSS8a	5/13/2010	Smith Creek (downstream), VA	Grab sample
PSS8b	6/14/2010	Smith Creek (downstream), VA	Grab sample
PSS9a	5/12/2010	Naked Creek, VA	Grab sample
PSS9b	6/15/2010	Naked Creek, VA	Grab sample
PSS10a	5/13/2010	Briery Branch, VA	Grab sample
PSS10b	6/15/2010	Briery Branch, VA	Grab sample
PSS11a	5/13/2010	Smith Creek (upstream), VA	Grab sample
PSS11b	6/14/2010	Smith Creek (upstream), VA	Grab sample
PSS12a	5/13/2010	Long Glade Creek, VA	Grab sample
PSS12b	6/14/2010	Long Glade Creek, VA	Grab sample
PSS13a	5/13/2010	Linville Creek, VA	Grab sample
PSS13b	6/14/2010	Linville Creek, VA	Grab sample
PSS14a	5/13/2010	Long Meadow Run, VA	Grab sample
PSS14b	6/14/2010	Long Meadow Run, VA	Grab sample
PSS15a	5/13/2010	Muddy Creek, VA	Grab sample
PSS15b	6/15/2010	Muddy Creek, VA	Grab sample
PSS16a	5/12/2010	Cooks Creek, VA	Grab sample
PSS16b	6/14/2010	Cooks Creek, VA	Grab sample
Dairy	9/28/2010	North Fork Shenandoah at Woodstock, VA	Grab sample
LF1a	10/15/2008	WWTP1 effluent, Rappahannock river, Little Falls, VA	Grab sample
LF2a	10/15/2008	WWTP2 effluent, Rappahannock river, Little Falls, VA	Grab sample
LF3a	10/15/2008	WWTP3 effluent, Rappahannock river, Little Falls, VA	Grab sample
LF4a	10/15/2008	WWTP4 effluent, Rappahannock river, Little Falls, VA	Grab sample
SUS1	5/12/2009	Susquehanna river at Mahantango Access, PA	Grab sample
SUS2	5/13/2009	West Branch Susquehanna river, PA	Grab sample
Plate 2			
SUS5	4/21/10-6/1/10	Juniata River, Newport, PA	POCIS
SUS6	4/21/10-6/1/10	Susquehanna River at Mahantango Creek, PA	POCIS
DE1	4/19/10-6/3/10	Delaware River, Yardley, PA	POCIS
AL1	4/28/10-6/7/10	Allegheny River, Kittanning, PA	POCIS
PSS16	5/12/10-6/14/10	Cooks Creek, VA	POCIS
PSS09	5/12/10-6/15/10	Naked Creek, VA	POCIS
PSS15	5/13/10-6/15/10	Muddy Creek, VA	POCIS
PSS10	5/13/10-6/15/10	Briery Branch, VA	POCIS
PSS12	5/13/10-6/14/10	Long Glade Creek, VA	POCIS
PSS11	5/13/10-6/14/10	Smith Creek (upstream), VA	POCIS
PSS13	5/13/10-6/14/10	Linville Creek, VA	POCIS
PSS14	5/13/10-6/14/10	Long Meadow Run, VA	POCIS
PSS08	5/13/10-6/14/10	Smith Creek (downstream), VA	POCIS
PSS06	5/13/10-6/14/10	Mill Creek, VA	POCIS
PSS02	5/14/10-6/14/10	Gooney Run, VA	POCIS
PSS07	5/14/10-6/14/10	Hawksbill Creek, VA	POCIS
PSS03	5/14/10-6/14/10	Passage Creek, VA	POCIS
PSS05	5/14/10-6/14/10	Stony Creek (downstream), VA	POCIS
PSS04	5/14/10-6/14/10	Stony Creek (upstream), VA	POCIS
FAB	6/1/2010	Blank	POCIS
1FB	6/1/2010	Blank	POCIS

2FB	6/1/2010	Blank	POCIS
3FB	6/1/2010	Blank	POCIS
4FV	6/1/2010	Blank	POCIS
5FB	6/1/2010	Blank	POCIS
6FB	6/1/2010	Blank	POCIS
7FB	6/1/2010	Blank	POCIS
8FB	6/1/2010	Blank	POCIS
9FB	6/1/2010	Blank	POCIS
10FB	6/1/2010	Blank	POCIS
11FB	6/1/2010	Blank	POCIS
12FB	6/1/2010	Blank	POCIS
13FB	6/1/2010	Blank	POCIS
14FB	6/1/2010	Blank	POCIS
15FB	6/1/2010	Blank	POCIS
16FB	6/1/2010	Blank	POCIS
17FB	6/1/2010	Blank	POCIS
18FB	6/1/2010	Blank	POCIS
19FB	6/1/2010	Blank	POCIS
BLANK	6/1/2010	Blank	POCIS
R38	9/20/2010	PA Erie (Pond H)	Grab sample
R39	9/28/2010	NJ Great Swamp (Hidden Valley Nursery)	Grab sample
R40	10/4/2010	VA Back Bay	Grab sample
R41	10/5/2010	VA Wilna Pond	Grab sample
R42	10/6/2010	VA Chandler's Mill Pond	Grab sample
R43	10/13/2010	MD Patuxent (Snowden Pond)	Grab sample
R44	10/13/2010	MD Patuxent (Cash Lake)	Grab sample
GL1	10/1/2010	Genesee River, NY	Grab sample
GL2W	10/1/2010	St. Louis River, Duluth, MN (WWTP effluent)	Grab sample
GL2P	10/1/2010	St. Louis River, Duluth, MN (PowerPlant effluent)	Grab sample
GL5	10/1/2010	Fox river, Green Bay, WI	Grab sample
GL4CSOa	10/1/2010	Swan Creek, Ohio	Grab sample
GL4CSOb	10/1/2010	Swan Creek, Ohio	Grab sample
GL4CSOc	10/1/2010	Swan Creek, Ohio	Grab sample
GL3a	10/1/2010	Detroit River, MI	Grab sample
GL3b	10/1/2010	Detroit River, MI	Grab sample
GL3c	10/1/2010	Detroit River, MI	Grab sample
R36	9/14/2010	Missisquoi River (Gander Bay & Goose Bay), VT	Grab sample
R37	9/16/2010	Lake Umbagog, MA	Grab sample

Supplementary Table S4. Additional samples tested for GFP-GR and GFP-AR translocation efficiency.

(b) Activity of the samples in the GFP-GR and GFP-AR translocation assays (P<0.01 and P<0.05, asterisks).

Samples	GR translocation		AR translocation	
	P<0.01	P<0.05	P<0.01	P<0.05
Plate 1				
R1	FALSE	FALSE	FALSE	FALSE
R2	FALSE	FALSE	FALSE	FALSE
R3	FALSE	FALSE	FALSE	FALSE
R4A	*	*	*	*
R4E	*	*	*	*
R5	FALSE	FALSE	FALSE	FALSE
R6	FALSE	FALSE	FALSE	FALSE
R7	FALSE	FALSE	FALSE	FALSE
R8	FALSE	FALSE	*	*
R9	FALSE	FALSE	*	*
R10	FALSE	FALSE	*	*
R13	FALSE	FALSE	FALSE	FALSE
R14	FALSE	FALSE	FALSE	FALSE
R15	FALSE	FALSE	*	*
R16	FALSE	FALSE	*	*
R16W	*	*	FALSE	*
R21	FALSE	FALSE	FALSE	FALSE
R22	FALSE	FALSE	FALSE	FALSE
R22W	FALSE	FALSE	FALSE	FALSE
R23	FALSE	FALSE	FALSE	FALSE
R24	FALSE	FALSE	FALSE	FALSE
R26	FALSE	FALSE	FALSE	FALSE
R27	*	*	FALSE	FALSE
R27W	FALSE	FALSE	FALSE	FALSE
R28	FALSE	FALSE	FALSE	FALSE
R29	FALSE	FALSE	FALSE	FALSE
R30	FALSE	FALSE	FALSE	FALSE
R31	FALSE	FALSE	FALSE	FALSE
R32 Broad	FALSE	FALSE	FALSE	FALSE
R32 CON	FALSE	FALSE	FALSE	FALSE
PSS2a	FALSE	FALSE	FALSE	FALSE
PSS2b	FALSE	FALSE	FALSE	FALSE
PSS3a	FALSE	FALSE	FALSE	FALSE
PSS3b	FALSE	FALSE	FALSE	FALSE
PSS4a	FALSE	FALSE	FALSE	FALSE
PSS4b	FALSE	FALSE	FALSE	FALSE
PSS5a	FALSE	FALSE	FALSE	FALSE
PSS5b	FALSE	FALSE	FALSE	FALSE
PSS6a	FALSE	FALSE	FALSE	FALSE
PSS6b	FALSE	FALSE	*	*
PSS7a	FALSE	FALSE	FALSE	FALSE

PSS7b	FALSE	FALSE	FALSE	FALSE
PSS8a	FALSE	FALSE	FALSE	FALSE
PSS8b	FALSE	FALSE	FALSE	FALSE
PSS9a	FALSE	FALSE	FALSE	FALSE
PSS9b	FALSE	FALSE	FALSE	FALSE
PSS10a	FALSE	FALSE	FALSE	FALSE
PSS10b	FALSE	FALSE	FALSE	FALSE
PSS11a	FALSE	FALSE	FALSE	FALSE
PSS11b	FALSE	FALSE	FALSE	*
PSS12a	FALSE	FALSE	FALSE	FALSE
PSS12b	FALSE	FALSE	*	*
PSS13a	FALSE	FALSE	FALSE	FALSE
PSS13b	FALSE	FALSE	*	*
PSS14a	FALSE	FALSE	FALSE	FALSE
PSS14b	FALSE	FALSE	*	*
PSS15a	FALSE	FALSE	*	*
PSS15b	FALSE	FALSE	FALSE	FALSE
PSS16a	FALSE	FALSE	FALSE	FALSE
PSS16b	FALSE	FALSE	*	*
Dairy	FALSE	FALSE	FALSE	FALSE
LF1a	FALSE	*	*	*
LF2a	*	*	*	*
LF3a	*	*	FALSE	FALSE
LF4a	*	*	FALSE	*
SUS1	FALSE	FALSE	FALSE	FALSE
SUS2	FALSE	FALSE	FALSE	FALSE
Plate 2				
SUS5	*	*	*	*
SUS6	*	*	*	*
DE1	*	*	*	*
AL1	*	*	*	*
PSS16	*	*	*	*
PSS09	*	*	*	*
PSS15	*	*	*	*
PSS10	*	*	*	*
PSS12	*	*	*	*
PSS11	*	*	FALSE	*
PSS13	*	*	*	*
PSS14	*	*	*	*
PSS08	*	*	FALSE	*
PSS06	*	*	*	*
PSS02	*	*	*	*
PSS07	*	*	*	*
PSS03	FALSE	FALSE	FALSE	FALSE
PSS05	*	*	*	*
PSS04	*	*	FALSE	FALSE
FAB	FALSE	FALSE	FALSE	FALSE

1FB	FALSE	FALSE	FALSE	FALSE
2FB	FALSE	FALSE	FALSE	FALSE
3FB	FALSE	FALSE	FALSE	FALSE
4FV	FALSE	FALSE	FALSE	FALSE
5FB	FALSE	FALSE	FALSE	FALSE
6FB	FALSE	FALSE	FALSE	FALSE
7FB	FALSE	FALSE	FALSE	FALSE
8FB	FALSE	FALSE	FALSE	FALSE
9FB	FALSE	FALSE	FALSE	FALSE
10FB	FALSE	FALSE	FALSE	FALSE
11FB	FALSE	FALSE	FALSE	FALSE
12FB	FALSE	FALSE	FALSE	FALSE
13FB	FALSE	FALSE	FALSE	FALSE
14FB	FALSE	FALSE	FALSE	FALSE
15FB	FALSE	FALSE	FALSE	FALSE
16FB	FALSE	FALSE	FALSE	FALSE
17FB	FALSE	FALSE	FALSE	FALSE
18FB	FALSE	FALSE	FALSE	FALSE
19FB	FALSE	FALSE	FALSE	FALSE
BLANK	FALSE	FALSE	FALSE	FALSE
R38	FALSE	FALSE	FALSE	FALSE
R39	FALSE	FALSE	FALSE	FALSE
R40	FALSE	FALSE	FALSE	FALSE
R41	FALSE	FALSE	FALSE	FALSE
R42	FALSE	FALSE	FALSE	FALSE
R43	FALSE	FALSE	FALSE	FALSE
R44	FALSE	FALSE	FALSE	FALSE
GL1	FALSE	FALSE	FALSE	FALSE
GL2W	*	*	FALSE	*
GL2P	FALSE	FALSE	FALSE	FALSE
GL5	FALSE	FALSE	FALSE	FALSE
GL4CSOa	FALSE	*	*	*
GL4CSOb	*	*	*	*
GL4CSOc	FALSE	FALSE	*	*
GL3a	FALSE	FALSE	FALSE	FALSE
GL3b	FALSE	FALSE	FALSE	FALSE
GL3c	*	*	FALSE	FALSE
R36	FALSE	FALSE	FALSE	FALSE
R37	FALSE	FALSE	FALSE	FALSE
